# Applications of human organoids in the personalized treatment for digestive diseases

**DOI:** 10.1038/s41392-022-01194-6

**Published:** 2022-09-27

**Authors:** Qinying Wang, Fanying Guo, Yutao Jin, Yanlei Ma

**Affiliations:** 1 Department of Colorectal Surgery, Fudan University Shanghai Cancer Center, Shanghai, China; 2 Department of Oncology, Shanghai Medical College, Fudan University, Shanghai, China; 3 School of Clinical Medicine, Shanghai Medical College, Fudan University, Shanghai, China

**Keywords:** Gastrointestinal diseases, Molecular biology

## Abstract

Digestive system diseases arise primarily through the interplay of genetic and environmental influences; there is an urgent need in elucidating the pathogenic mechanisms of these diseases and deploy personalized treatments. Traditional and long-established model systems rarely reproduce either tissue complexity or human physiology faithfully; these shortcomings underscore the need for better models. Organoids represent a promising research model, helping us gain a more profound understanding of the digestive organs; this model can also be used to provide patients with precise and individualized treatment and to build rapid in vitro test models for drug screening or gene/cell therapy, linking basic research with clinical treatment. Over the past few decades, the use of organoids has led to an advanced understanding of the composition of each digestive organ and has facilitated disease modeling, chemotherapy dose prediction, CRISPR-Cas9 genetic intervention, high-throughput drug screening, and identification of SARS-CoV-2 targets, pathogenic infection. However, the existing organoids of the digestive system mainly include the epithelial system. In order to reveal the pathogenic mechanism of digestive diseases, it is necessary to establish a completer and more physiological organoid model. Combining organoids and advanced techniques to test individualized treatments of different formulations is a promising approach that requires further exploration. This review highlights the advancements in the field of organoid technology from the perspectives of disease modeling and personalized therapy.

## Introduction

The digestive system, a continuous anatomical structure composed of multiple organs, is responsible for swallowing and digesting food, absorbing nutrients, and discharging residual wastes.^1,2^ The accumulation of many external stimuli and genetic mutations contributes to the emergence and progression of digestive diseases, including infectious, inflammatory, and malignant diseases.^3–8^ The morbidity and mortality of certain diseases are increasing annually, despite the constant updating of treatments.^9–16^ Therefore, it is of great significance and urgency to clarify the etiology of digestive diseases and find new and more effective treatments. Achieving the above goals will require two foundations: on the one hand, omics technologies and bioinformatics analysis are necessary to find correlations, and many achievements have been made in this field,^4,17–27^ on the other hand, reliable models can be used to reveal causal relationships, find molecular targets, and test therapeutic strategies. Ultimately, and most importantly, basic research can be translated into clinical research to benefit patients.

The human digestive system is not directly accessible and translating the rapidly evolving preclinical knowledge associated with diagnostics and therapeutic interventions is not an easy task. Differences in the anatomy, biological processes and cell-type-specific expression patterns of the human digestive system make animal models a suboptimal choice to mimic the occurrence and treatment of human digestive diseases.^28–31^ The limitations of cell line models established from the human digestive system are as follows: primary cells established from the original tissue have a short lifespan and require strict culture conditions; if immortal or cancerous cells are used, they will lose the complex characteristics of primary tissue after a long period of artificial culture on plastic plates with non-physiological media and may not respond to interventions in the same way as host tissue would.^32–34^ These defects mean that, while mouse models and cell lines have provided some understanding and insight into digestive system evolution and disease over the years, the failure rate of clinical translation has been quite high.^35–38^ Over the past decades, many approaches have been used in the attempt to generate models of the human digestive system in vitro.^34^ The development of organoid culture was a major breakthrough.^39,40^ Organoids recapitulate many biological features of human organs, including tissue heterogeneity, spatial distribution characteristics of different cells, cell-cell and cell-matrix interactions, and some functions arise from tissue-specific cells.^41–43^ Organoids are more representative of in vivo physiology and bridge the gap in existing model systems with a stable system adapted to a wide range of cultures and manipulations.^44–47^ Different cell types of stem cell-derived organoids contain complex cues that regulate tissue formation, giving them a unique potential in the study of biological phenomena related to tissue development and stem cell differentiation.^48–50^ Importantly, adult stem cell (AdSC)-derived organoids from individual patients can be expanded and preserved for therapeutic testing, be used to preserve tissue properties to achieve optimal outcomes for each patient, even be served as preclinical tools for drug screening.^41,50–52^

**Fig. 1:**
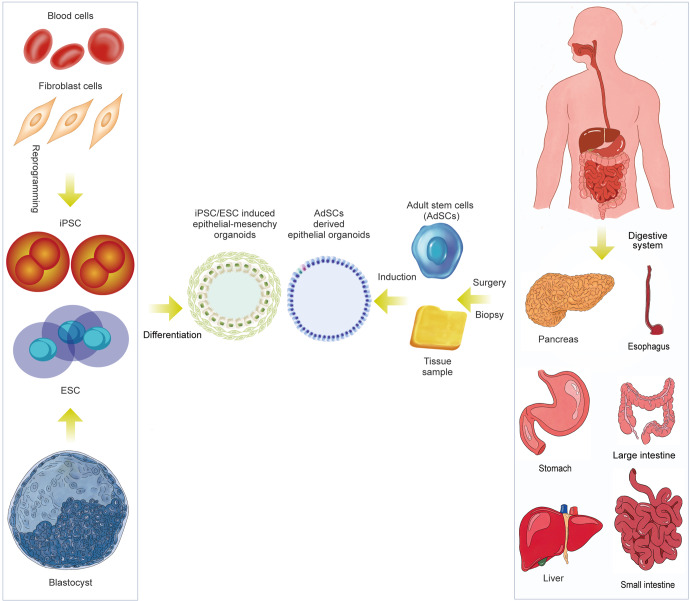
Schematic diagram summarizing the generation of digestive organoids and the manner of cell differentiation. Organoids derived from isolated epithelial stem cells and tissue samples, reprogramming of skin fibroblasts and blood cells into pluripotent stem cells (PSCs). (Induction involves germ-layer specification (endoderm) and subsequent induction and maturation by specific growth factor combinations.)

In this review, we summarize the various in vitro digestive organoid models that have been created and the compositional differences between digestive organoids generated from AdSCs and induced pluripotent stem cells (iPSCs) (Fig. 1). In addition, we summarize how they reshape or deepen our understanding of the occurrence and characteristics of digestive disease, stem cell function and regeneration, host-pathogen interactions, and the exploration and application of personalized therapy. We also discuss the current shortcomings of organoid models and directions for future improvement.

## Digestive organoids: self-organizing systems of organs ex vivo

Organoids are generated from various types of stem cells, which are induced to form microscopic cell clusters and cultured in vitro as 3D structures; these clusters are also known as “mini-organs” because they mimic the in vivo structures and functions of the corresponding organs.^53^ Organoid formation relies on decades of research into stem cell fate determination, which is tightly coordinated by the Wnt/Notch/EGF/BMP pathway.^54,55^ Inspired by these studies, Cleves’ team established the first mouse gut organoid in 2009 by isolating Lgr5^+^ stem cells ex vivo with a combination of EGF, Wnt3a, and the BMP inhibitors Noggin or R-spondin-1 in a basal medium under a dome of matrigel.^56^ Their team subsequently modified the culture medium in 2011 to obtain human colonic organoids.^57^ Intestinal organoids are commonly termed “enteroids” when they originate from the small intestine and “colonoids” when they originate from the colon.^58^ The establishment of mouse- or human-derived epithelial enteroids and colonoids relies on the utilization of different culture media. For example, media containing R-spondin1, EGF, and Noggin is required for mouse intestinal organoids and does not vary with diverse proliferation and differentiation conditions. Additional Wnt3a results in indefinite growth of mouse intestinal organoids devoid of a functionally intact Wnt-secreting niche, necessitating the addition of exogenous Wnt3a to the expansion medium and the exclusion of Wnt3a from the differentiation medium. The long-term maintenance of human intestinal organoids requires more pathways, such as inhibition of the TGFβ and P38 MAPK pathways by the chemical inhibitors A83-01 and SB202190 or the addition of insulin-like growth factor 1 (IGF1) and fibroblast growth factor 2 (FGF2). Over decades of research, with continuous testing of combinations of nutritional factors, organoid models of the human digestive system have been developed into the more mature in vitro models, replicating the human esophagus, stomach, liver, pancreas, etc.^59–62^ These organoids are obtained from ex vivo expanded resident tissue stem cells by recreating the stem cell niche. Although each organ is unique in structure and cellular composition, organoids derived from those stem cells are very similar in terms of mitogenic factors, culture conditions, and final structure. The Wnt pathway activator Wnt-3A or R-Spondin, the BMP pathway antagonist Noggin, EGF or FGF, and TGF-β inhibitors are often needed, as well as other factors depending on the tissue origin. These multicellular organoids consist exclusively of simple hyperpolarized epithelial cells tightly surrounding the central lumen and outwardly projecting crypt-like structures.^63^

In addition to adult stem cell-derived organoids, digestive organoids can also be obtained from pluripotent stem cells (PSCs), including iPSCs and ESCs, in a step-by-step manner by mimicking embryonic development after implantation under a complex, coordinated set of specifications to determine the formation of morphological features.^64,65^ Likewise, PSC-derived organoids were first induced to develop into intestinal organoids through a gradual series of steps, in which activin-A first induced and defined endoderm formation and FGF/Wnt induced posterior endoderm patterning, subsequent hindgut specification and morphogenesis. After 4 weeks of culture, induced human intestinal organoids (iHIOs) were induced to express an intestinal stem cell marker, and they formed a polarized columnar epithelium with villus-like structures and an overall morphology similar to that of the human intestinal epithelium. iHIOs mimic the development of intestinal morphology and molecules. Later, PSC-derived organoids were also described for the large intestine.^65,66^ In the same manner as the endoderm, differentiation into the foregut, midgut and hindgut is finely regulated by a combination of factors. Furthermore, according to the fine-tuning regulation of the combination of factors obtained by accumulating embryonic development information on each organ, the foregut forms the esophagus, stomach, liver and pancreas, and the hindgut forms the large intestine. Thus, PSCs can form various digestive organs, such as the esophagus, stomach, intestine, liver, and pancreas, under conditions that closely mimic early patterning and morphogenesis with various specific medium components.^67–73^ In contrast to adult stem cells, PSCs are pluripotent, and various cellular components of the three germ layers can be obtained through different induction programs. In addition to the epithelial system, PSC-derived digestive organoids also contain a layer of mesenchymal cells.^74,75^ Hence, digestive organoids have extensive applications in various areas. Comprehensive protocols for establishing digestive organoids have been published, which enable the long-term expansion and passaging of organoids that can be cultured as desired for drug screening or microbial infection and mechanistic studies.^76–86^

## The application of organoids

**Fig. 2:**
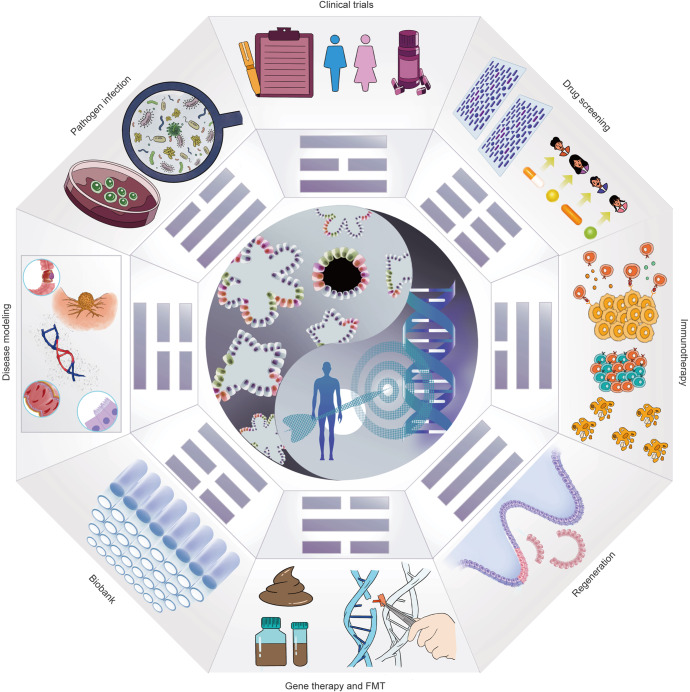
Potential applications of organoid models in precision medicine. As a versatile in vitro model, organoids can cover many applications from disease modeling to clinical trials and achieve the purpose of precise and personalized treatment. The scope of organoid research includes (1) Disease modeling. (2) Pathogenic infection. (3) Biobanking of organoids. (4) Clinical trials. (5) Stem cell function and regeneration (delivery of organoids to repair intestinal epithelium or activate the repair program of endogenous stem cells). (6) Drug screening (patient-specific high-throughput screening and discovery). (7) Immunotherapy. (8) Gene therapy and FMT

As organoid technology opens new areas of biomedical research, the establishment of long-term in vitro models of various digestive organs has increased our knowledge of digestive diseases, biobanking, clinical trials, pathogen infections, drug screening, stem cell function and regeneration, immunotherapy, gene therapy, testing for fecal microbiota transplantation (FMT), etc., all of which contribute to the development of personalized medicine (Fig. 2).

### Digestive system disease modeling

The pathogenesis of digestive system diseases is complex, occurring as a series of consequences of the interweaving and interaction of genes and the environment, and this process is the sum of infectious diseases, inflammatory diseases, and malignant tumors.^87–89^ Abnormal expression or mutation of genes such as TP53, SMAD4 and PIK3CA mediate the occurrence and progression of diseases. In terms of environmental factors, these digestive organs tend to be prone to pathogen infections or external liquid and gaseous irritation. Notably, important aspects of anatomy and compartmentalization vary widely between species.^36,37,90^ Therefore, human digestive diseases can be studied most effectively by using human model systems. Organoid models derived from the digestive system can closely mimic these diseases due to their good representation of the cell composition and molecules characteristics of human organs.^41^ The simulation of digestive diseases by organoids can be realized mainly through two channels. First, the organoids of patients can be generated from the tissues obtained from surgery or biopsy to construct biobanks of patient-derived organoids (PDOs). PDOs have been shown to preserve their clinical markers, histomorphology, driver mutations, and even molecular and metabolite heterogeneity, which assures that organoids can respond realistically to in vivo treatments.^91^ Another approach is to mimic disease onset and progression by manipulating the expression of disease-causing genes or to generate mutation points by combining gene editing techniques. This method can be divided into two types: gene editing in organoid models derived from healthy/paracancerous tissue, and gene editing in organoid models derived from iPSCs. Each type has its own advantages: organoids derived from adult tissues retain the epigenetic and genetic information of adult tissues, making them more similar to the physiological state of human organs; PSC cells avoid the complex phenotypic variation of organoids from different backgrounds, allowing a variety of complex genetic manipulations and multivariate observations. Furthermore, the organoid models are also used to simulate environmental disturbances, in order to observe how those pathogens interact with the digestive organs and mediate barrier disruption, inflammation and immune responses, the genetic mutations, that ultimately cause a range of digestive diseases. At present, organoids are commonly researched in the medical field by extracting diseased tissues from patients and generating organoids to study the proportion of disease mutations that the organoids can retain and to what extent they can simulate patients’ responses to various clinical treatment strategies.^51,92^ Intervening in expression or activity of one or several genes or protein by a series of molecular means to study the association between corresponding genes and the occurrence or progression of diseases has been carried out mostly in the field of basic research.^47,93,94^ Together, these two advances form the basis of our current understanding of how organoids mimic digestive system disease and reveal its occurrence and progression.

### Intestinal disease

The intestine is one of the largest vital organs of the human body, integrating many complex epithelial, immune, lymphatic, vascular, and nervous components to perform digestive and endocrine functions and regulate digestion, metabolism, and feeding behaviors.^41,95,96^ It can be divided into two segments, the small intestine and the large intestine. In the past decade, the generation of intestinal organoids has provided a rich reference for understanding intestinal pathology.^97–99^ Organoid models can effectively mimic the state of intestinal diseases, including motility disorders, malabsorptive diarrhea, inflammation, infection, and cancer.^40,75,95,100^ Microvillus inclusion disease (MVID) is a congenital disease of the intestinal epithelium that causes malnutrition and is characterized by diffuse villus atrophy or absence of apical microvilli.^101,102^ MVID cases are caused by mutations in either myosin 5B (MYO5B) or syntaxin-3.^103^ Loss of the microvillus phenotype was reported in a patient-tissue-derived organoid model.^104,105^ Deletion of MYO5B impairs precursor differentiation into organoids and partially mediates Wnt/Notch imbalance, and Notch inhibition and/or LPA treatment could be an effective treatment for MVID.^106^ Diacylglycerol-acyltransferase 1 (DGAT1) dysfunction often leads to another rare, intractable form of malabsorption and diarrheal disease that appears early in life.^107^ The main role of DGAT1 is to catalyze the conversion of diglycerides and fatty acyl-CoA to triglycerides, and DGAT1 deficiency can lead to fat intolerance and protein-losing enteropathy.^108^ Intestinal organoids produced from tissues of DGAT1 mutant patients showed decreased lipid catabolism and increased cell death after oleic acid treatment. Knockdown of DGAT1 in normal organoids can also replicate this phenotype, which essentially recapitulates and reflects the fat intolerance and intestinal damage caused by DGAT1 deficiency in patients.^109^ Multiple intestinal atresia (MIA) is a rare disease with bowel obstruction and uniform calcification in the abdominal cavity, caused by a deficiency of the tetratricopeptide repeat domain 7 A gene (TTC7A).^110,111^ Organoids formed from MIA patient tissue have inside-out apicobasal polarity, which is rarely observed in long-term culture; however, this reversal can be normalized by pharmacological inhibition of Rho kinase.^112^ Cystic fibrosis (CF) is a common, fatal, multisystem genetic disorder that presents as a chronic disease with an altered gut environment.^113^ It is a disease caused by impaired epithelial anion transport owing to mutations in the gene encoding cystic fibrosis transmembrane regulator protein (CFTR), resulting in impaired fluid regulation and pH imbalance in multiple organs.^114,115^ CFTR-dependent secretory responses can be replicated by intestinal organoids from CF patients, and it was found that increased ATP treatment of these organoids with forskolin causes CFTR-mediated chloride channel opening and induces fluid secretion from epithelial cells, resulting in swelling of intestinal organoids.^116^ Inflammatory bowel diseases (IBDs) come in two forms, known as ulcerative colitis (UC) and Crohn’s disease (CD); these debilitating chronic inflammatory disorders are consequences of genetic predisposition combined with accumulated dysbiosis of the gut microbiota, which mediates uncontrolled immune responses and impaired structural function of the gut epithelium.^117–119^ Organoid models can also be used to interrogate complex somatic cells in UC tissue as they evolve to adapt to the chronic inflammatory microenvironment.^117,120–123^ Intestinal organoids harvested from CD patients can recapitulate multiple patterns of disease evolution, including higher organoid reorganization capacity, DNA methylation, and changes in transcriptome and stem cell marker gene profiles.^123–125^ Despite a comparable mutational signature, the number of single nucleotide variants (SNVs) in UC organoids from the same patient was slightly higher than that in paired control organoids, which essentially mimics the finding that epithelial cells in UC have an increased number of mutations under inflammatory conditions in vivo.^120,126^ Human iPSCs from colonic fibroblasts were isolated from UC patients, followed by targeted differentiation into organoids; these organoids fully reproduced the histological and functional features of primary colonic tissue, including insufficient secretion of acidic mucus, abnormal adherent junctions in the epithelial barrier, and overexpression of the CXCL8/CXCR1 axis.^121^ In addition, organoids can provide a good model for the simulation of colorectal cancer progression.^127–130^The occurrence and development of Colorectal Cancer (CRC) is a malignant tumor and a heterogeneous disease involving a series of genomic alterations.^131–134^ The occurrence and development of CRC is a multifactorial, multi-pathway process that follows a progression from normal to adenoma to adenocarcinoma to liver metastasis of adenocarcinoma. Organoids derived from colonic tissue have been used to model CRC, and these PDOs recapitulate the somatic copy number alteration (SCNA) and mutational spectrum found in CRC, with ~80% of the mutations in primary tumor tissue being identified in the corresponding organoids.^135^ Metastasis is the main cause of death in CRC; patient-derived paired primary and metastatic tumor organoids have been selected as CRC metastasis models, which can be used for the discovery and detection of potential prognostic biomarkers and therapeutic targets of CRC. Despite shared driver mutations between primary and metastatic tumors, metastatically derived organoids after xenotransplantation exhibit higher metastatic capacity.^136^ When the multimutated CRC genes APC, KRAS, SMAD4, TP53 and PIK3CA were introduced into normal human intestinal organoids using CRISPR-Cas9 genome editing technology, the organoids could simulate the progression of CRC and gradually transform into invasive glandular tissues in vivo.^137^ Gene-edited Apc/KrasG12D/Trp53 organoids were transplanted into the distal colon and subsequently found to metastasize to the liver.^138^ Deletion of MLH1 from colonic epithelium-derived normal organoids using CRISPR-Cas9 technology mimics features of MSI-driven CRC.^139^ Several newly discovered causative genes for colorectal cancer, such as the protein tyrosine kinases (PTKs) BMX and HCK observed in adenoma precursor cells, have been suggested as potential drivers of adenomas. When these two genes were exogenously expressed in human intestinal organoids, an increase in BMX or HCK was observed to significantly improve the number and size of organoid hyperplasia within the organoid lumen, as well as multiple polyp bud protrusions and wall hyperplasia.^140^ In addition to the role of gene mutation, chromosomal instability is also one of the pathological causes of CRC.^141^ When combined with other interventions, xenografted mutant organoids with certain chromosomal rearrangements, including reversals and deletions affecting R-spondin2, form flat serrated lesions similar to sessile serrated adenoma in mice.^142^

### Gastric disease

The stomach is composed of ordered epithelium, and the invaginated gastric unit contains acid-secreting parietal cells, mucous pit cells, enzyme-secreting primary cells, proliferating cells, and intermediate cell populations.^143^ Gastric cancer (GC) is a heterogeneous malignancy of the digestive system with complex and diverse mutated genes and pathogenic mechanisms.^144^ GC can be divided into four subtypes according to molecular typing: the chromosomally unstable (CIN) subtype, frequently featuring RTK-RAS pathway amplification and mutated in TP53; the microsatellite instability (MSI) subtype, with a hypermutated phenotype; the genomically stable (GS) subtype, with alterations in RHOA/CDH1 and manifesting as diffuse tumor morphology; and the Epstein-Barr virus (EBV)-positive subtype, which displays frequent CDKN2A dysfunction and PIK3CA mutations.^145–147^ The established GC organoid biobank essentially covers all four GC subtypes and captures unique molecular profiles of dysregulated genes that can be used to guide unique therapeutic targets for GC subtypes.^148–150^ GC organoids retain cellular markers and tumor histopathological classification, and GC-associated gene mutations and copy number variations (CNVs) are also very similar in PDOs and corresponding primary tumors.^151^ Some heterogeneity was noted in lymph node metastatic GC organoids; the organoids from primary tumor histology showed diffuse growth, and organoids from lymph node metastatic tumors showed glandular shapes.^152^ The genotype-phenotype association can be validated by the phenotypic features of gene-edited gastric-like organoids carrying multiple gastric cancer mutations, such as the migration and diffuse appearance of gastric organoids after CDH1 knockout.^148^ GC metastasis can also be replicated by establishing an orthotopic carcinoma organoid transplantation model and identifying the essential characteristics of stem-like TR-LGR5^+^ cells in the persistence and metastasis of GC.^153^

### Esophagus diesease

The esophagus consists of four layers: inner mucosa, the underlying supporting submucosa, the muscularis propria, and the epithelium and adventitia; it acts as an anatomical conduit for the transport of food from the pharynx to the stomach.^38,154^ Prolonged exposure to gastroesophageal reflux can lead to Barrett’s esophagus (BE); the transition from normal squamous epithelium to abnormal specialized columnar epithelium is also known as intestinal metaplasia in the esophagus; BE is increasing in incidence and is a potential risk for esophageal adenocarcinoma (EAC).^155–157^ BE organoids contain periodic acid-Schiff-positive goblet cells, a hallmark of BE.^158^ Esophageal cancer is a malignancy with high morbidity and mortality. There are two major histological subtypes, EAC and esophageal squamous cell carcinoma (ESCC).^159,160^ EAC cells are morphologically similar to the secretion-producing glandular cells of the intestinal mucosa, whereas ESCC cells exhibit varying degrees of squamous cell differentiation. Organoids obtained from EAC recapitulated patient tumor histology, including p53 status and apical/basal polarity.^161^ Furthermore, organoids obtained from EAC also showed the same consistency between driver somatic mutational events and genome-wide mutational signatures as in the original tumor tissue, including mutations of CDKN2A and PIK3CA.^161,162^ The characteristic clonal heterogeneity of EACs, which contributes to chemotherapy resistance and poor patient survival, can be retained in the resulting organoids.^163^ Established ESCC organoids from biopsy samples show consistency with the original tumors.^164^

### Pancreatic disease

The main function of the pancreas is to facilitate digestion and metabolism. The pancreas consists of three main cell types, namely, acinar cells, ductal cells, and endocrine cells that are responsible for stabilizing blood sugar; dysfunction of this organ can lead to diabetes and pancreatic cancer.^165–167^ Pancreatic ductal adenocarcinoma (PDAC) is the most lethal common malignancy, with poor prognosis and high mortality, and accounts for 90% of pancreatic cancers; PDAC is also one of the most drug-resistant cancers due to the extensive heterogeneity of mutations and the dense stromal environment.^168–170^ Pancreatic cancer organoids are derived from primary tumors and metastatic tumors, retaining characteristics of primary malignancies; furthermore, organoids from peritoneally disseminated nodules not only retain proliferative capacity but also form peritoneal tumors with features of metastatic pancreatic cancer.^171^ Seventy percent of PDAC organoids are characterized by the classical or progenitor subtype, and approximately 30% are characterized by the basal or quasi-mesenchymal subtype; commonly mutated genes, such as TP53, KRAS, SMAD4 and CDKN2A, and uncommonly mutated genes, such as PIK3CA, MAP2K1 and ERBB2, were found in PDAC-generated organoids.^172,173^ When CRISPR-Cas9 genome editing technology is used to modify normal organoids to mimic the pancreatic disease, such as editing PDAC driver genes in PDAC organoids; mediating KRASG12V and ERBB2 mutations; and inactivating TP53, CDKN2A and SMAD4, and these mutant organoids develop into tumors that resemble pancreatic intraepithelial neoplasia after orthotopic xenografting in immunodeficient mice.^174,175^ Pancreatic duct and acinar organoids derived from human iPSCs can reproduce the characteristics of the neonatal exocrine pancreas, and the PDAC-related oncogene GNASR201C is more efficient at inducing cystic growth when expressed in ducts, whereas KRASG12D is more efficient at modeling cancer in vivo when expressed in acinar cells.^176^

### Liver diesease

The liver consists of parenchymal and nonparenchymal hepatocytes, organized into functional units called hepatic lobules; this organ carries out a variety of functions, such as digestion, detoxification, and metabolism.^177^ Mutations in the SERPINA1 gene mediate alpha-1-antitrypsin (A1AT) dysfunction, and patients with this mutation may develop chronic liver damage.^178,179^ Liver organoids from A1AT-deficient patients result in decreased A1AT secretion and increased endoplasmic reticulum stress due to misfolding of the mutated A1AT protein.^180^ Mutations in the Notch signaling pathway can cause Alagille syndrome, which is characterized by partial or complete atresia of the bile ducts and the bile duct function that is prevented from being established.^181^ Established liver organoids from Alagille syndrome patients treated with cholangiocyte differentiation mediators fail to upregulate bile markers.^182^ Fatty liver disease is attributed to a variety of causes, including an obese diet, a sedentary lifestyle, and the prevalence of metabolic disorders.^183,184^ Human hepatocyte-differentiated organoids and liver-derived intrahepatic cholangiocyte organoids can recapitulate some pathologies of fatty liver disease, such as lipid accumulation and mitochondrial damage.^185^ Insufficient lysosomal acid lipase activity leads to Wolman disease, a massive accumulation of lipids in liver cells with fatal steatohepatitis and fibrosis.^186,187^ Wolman disease-specific induced pluripotent stem cells generate liver organoids that recapitulate severe steatohepatitis and fibrosis.^187^ Nonalcoholic fatty liver disease can lead to steatohepatitis, which eventually leads to liver fibrosis, cirrhosis, and hepatocellular carcinoma (HCC).^188^ Human iPSC-derived liver organoids (HLOs) contain multiple cell types, such as stellate cells, hepatocytes, and kupffer-like cells, and have succeeded in recapitulating some features of steatohepatitis, including inflammation, lipid accumulation and fibrotic phenotypes, after oleic acid treatment.^187^ In a drug-induced organoid model of liver fibrosis, these organoids exhibited fibrotic features, such as human stellate cell activation, collagen secretion and deposition, when exposed to profibrotic compounds.^189^ Organoids generated from patients with primary sclerosing cholangitis showed changes in the expression of genes responsible for primary sclerosis.^190,191^ Human hepatocyte organoids were cocultured with mesenchymal cells from fetal liver tissue to recapitulate the pathophysiology of alcohol-related liver disease; hepatocyte-like cells in these organoids underwent oxidative stress, increased reactive oxygen species, and accumulated intracellular lipid droplets to induce steatosis and release inflammatory mediators after exposure to alcohol.^192^ Most primary liver cancers have low curative effects and high recurrence rates and can be divided into the hepatocellular carcinoma, cholangiocarcinoma and combined HC-CCA types.^193^ The generation of liver cancer (LC) organoids summarizes the molecular profiles of the corresponding tumor sources, including the PDO lineage of HCC, cholangiocarcinoma (CCC), and HCC/CCC combination; furthermore, lung metastases were found after transplantation into immunodeficient mice.^194,195^ Biopsy-derived HCC/CCC organoids also matched the mutational spectrum of primary tumor tissue and retained common driver mutations typical of HCC/CCC in ARID1A, TP53 and TSC1.^195^ In the study of the molecular mechanisms and related risk factors for primary liver cancer, there are also cases of organoids. For example, knockdown of arginine methylation transcription factor (PRMT6) in patient-derived nontumor liver organoids with CRISPR-Cas9 was found to increase cellular resistance to molecularly targeted drugs and chemotherapy; PRMT6 overexpression in HCC organoids attenuates tumor cell migration and invasion.^196^ Depletion of the tumor suppressor BAP1 in normal human cholangiocyte organoids results in impaired chromatin accessibility, loss of multiple epithelial features, and increased motility.^197^ Axin2 is highly expressed in HCC organoids, while RNF43/ZNRFR3 is deficient; in vitro genetic intervention produced lipid droplet aggregation in RNF43-mutated human hepatocellular carcinoma tumor organoids, whereas RNF43/ZNRF3-deficient hepatic epithelial organoids displayed not only lipid droplet aggregation but also reduced differentiation capacity.^198^

## Microecosystem research using digestive organoids

As previously mentioned, in addition to primary tissue extraction and gene editing to mimic digestive system disease, organoids can serve as a basis for reflecting a variety of complex environmental factors that regulate cell fate changes and disease development.^199,200^ Here, we discuss only the simulation of microbiota and viral infection in well-studied organoid systems.

### Microbiota and digestive disease

**Fig. 3:**
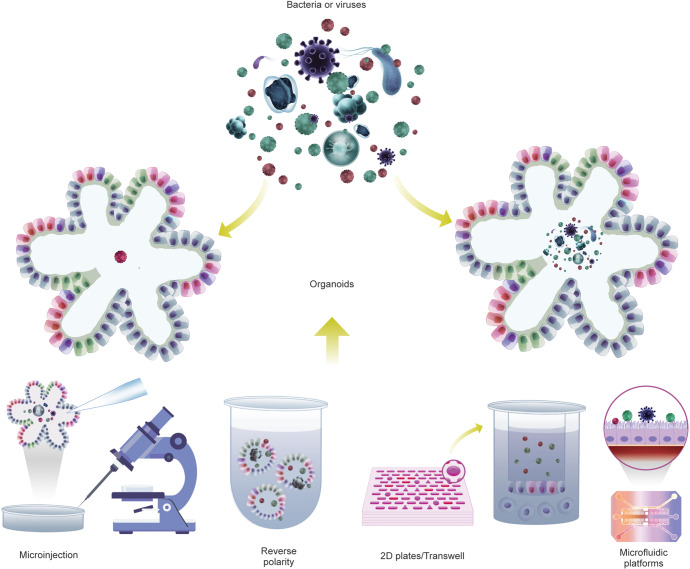
Using gut organoids to study the impact of microbiota. Methods of infecting organoids with bacteria: (1) Microinjection. (2) Transwell/Plate-based 2D monolayer cultures of organoids. (3) Reversal of cell polarity of organoids. (4) Organoids-on-a-chip

The microbiota shapes the chemical environment of the gut, the bioavailability of ingested substances, and the biological processes of tissue homeostasis; dysbiosis shift of the microbiota is expected to lead to diseases.^201,202^ Over the past decade, numerous studies have found that dysbiosis affects the occurrence and development of digestive system diseases.^203–209^ Nevertheless, numerous associations of microbes with those diseases remain correlative due to the difficulties of modeling the host-microbe relationships in a reductionist yet meaningful way that allows detailed mechanism analysis. Currently, there are two main approaches to exploring the impact of the microbiota on host physiopathology in mouse models, namely, germ-free animals and antibiotic regimens.^210,211^ Antibiotic treatment depletes the gut microbiota of mice with broad-spectrum antibiotic treatment. Antibiotics are usually dissolved in drinking water, provided ad libitum throughout treatment; therefore, actual doses administered may be unstable, so that the bacteria are not completely cleared from the treated mice, and levels of clearance vary between individuals. Thus, while antibiotic treatment provides an inexpensive and easy-to-use alternative to sterile models, the results obtained by these protocols have the potential for off-target drug effects, as well as incomplete or inconsistent ablation of microbes.^212–214^ Germ-free (GF) mice represent a system that allows the study of animals completely devoid of microbes, in which researchers introduce microbes into GF mice individually or sequentially and evaluate the effects of individual bacteria or known bacterial communities on host function impact.^204^ GF animal models are powerful tools for studying microbe-host interactions in health and disease.^204,215^ However, the high cost of GF mice and the relatively complex experimental manipulation techniques needed for these animals are barriers to the widespread adoption of this model for basic research. In addition, the species differences between animals and humans have always been an insurmountable gap. In particular, some microbes are unique to humans; therefore, conclusions from mouse models may not carry over. These dilemmas create the need for another set of model systems to help mimic the human traits that control the off-target effects of these bacteria and the perturbations of the aforementioned complexities. Organoid models derived from human tissue or cells are amenable to sterile culture methods and are a good alternative to GF mice. The 3D organoid model shows a closed, irregular luminal structure with most microbial-epithelial interactions taking place at the apical side of the epithelium. However, introducing the microbiome into the lumen of an organoid is not an easy task. There are several ways to do this (Fig. 3), including microinjection (A),^61,67,85,216,217^ 3D organoids grown with reversed polarity (B),^84,218,219^ organoid-derived fragments or epithelial monolayers (C),^220–224^ and microfluidic platforms (D).^225–229^ Each approach has its advantages and disadvantages (Table 1). Directly injecting microorganisms into the lumen of differentiated or undifferentiated 3D organoids, allowing microorganisms to contact the top of epithelial cells in 3D conditions, is a popular research method. However, microinjection requires a special setup and specialized techniques, and it can affect the readout when injected bacteria leak into the basolateral region, also coculturing obligate anaerobic bacteria for a long period of time is difficult due to the lack of sufficient oxygen in the lumen of organoids. 3D organoids growing with reversed polarity (the ‘apical-out’ orientation) expose the apical side of epithelial cells directly to the medium without matrix embedding, making it accessible to microorganisms. Continuous organoid cultures in prolonged suspension were observed to assess microbial-epithelial interactions and their consequences by adding microbes directly to the culture medium. Moreover, this approach enables easy testing of interactions with bacteria or their metabolites in a high-throughput setup under multiple conditions. However, since this method does not guarantee complete polarity reversal, distinguishing between apical and basolateral interactions is difficult. Another limitation of this approach is that the mucus can be easily washed out. Fragmented organoids and organoid-derived epithelial monolayers are another set of advancements enabled by linearization of 3D organoids into 2D systems to enhance the accessibility of the apical side. Organoids of various origins are divided into small fragments or single cells and then distributed in extracellular matrix-coated dishes or Transwell plates to form organoid-derived monolayers that differentiate into different epithelial cell lineages capable of producing mucus; microbes were added directly to the culture medium, and their interactions with the epithelial cells were observed. This relatively simple experimental setup can be used to compare a variety of conditions, both for culturing under aerobic conditions and for creating an anaerobic environment in the upper chamber of a Transwell plate. For example, the air-liquid interface (ALI) culture system provides a feasible mode for culturing obligate anaerobic bacteria. The apical cavity creates anoxic conditions for anaerobic bacteria, while the basal and lateral surfaces of the organoids are supplied with oxygenated medium. This system can also support longer-term maintenance of epithelial cell monolayers and expand the study of epithelial-mesenchymal interactions due to the mitigation of oxidative stress and the addition of potential stromal elements in ALI. However, these approaches have the disadvantage of losing the three-dimensional structure of the organoids. Culturing organoid monolayers on micro-structured collagen scaffold assemblies with crypt-like invaginations is an improvement that mimics the three-dimensional spatial features defining crypt-like and villus-like structures. However, maintaining the entire system in an anaerobic chamber is expensive, and replicating models can be time consuming. Organoids on microfluidic platforms are micro-engineered systems generated based on industrial computer microchips by microfabrication methods. Current microfluidic platforms include organoids-on-a-chip, HuMiX and GuMI. Microfluidic culture systems can enhance cellular microenvironments, replicate distinct mucus layers, mimic key features of the human gut, and monitor organoid metabolism. These systems have obvious advantages in studying host-microbe interactions and some complex biological functions in vitro. However, these models are expensive, requiring special training to operate and are difficult to use in high-throughput experiments. Each of these methods of delivering bacteria to organoids has both pros and cons. These methods are also constantly being optimized and improved, and efforts are being made to develop newer methods. Regardless of the specific method, however, these approaches have all facilitated the study of organoid-bacterial interactions and provided observational insights that advance our understanding of molecular mechanisms.

**Table 1:** Comparison of methods for introducing microorganisms into organoids

Method	Description	Advantages	Disadvantages
Microinjection^61,67,85,216,217^	Injecting microorganisms into the lumen of organoids using a microinjection device.	1. The method preserves the structural integrity of the organoids, and microorganisms only contact the apical side of organoids, providing a more realistic gastrointestinal simulation environment, especially for the anaerobic bacteria. 2. The entire process of microbe-organoid interactions can be observed, including initial interactions and early host responses. 3. Quantitative experiments can be performed by controlling the MOI. 4. This method has no special requirements for organoid culture conditions and can be applied for most 3D organoids.	1. This method requires a very specialized setup and is lacking of a standard procedure for different organoids. 2. The manual nature of the microinjection process makes it difficult to apply to high-throughput experiments, and the sequential injections resulted in asynchrony of experimental exposures. 3. The leakage of injected microorganisms toward the basolateral side can influence the readout, and the closed lumen may cause nutrient and oxygen consumption and metabolites accumulation. 4. Injections of small volumes of material are often imprecise, and differences in organoid size as well as luminal contents may cause uneven distribution of the injected material.
Organoid-derived fragment or epithelial monolayers^220–224^	Linearizing 3D Organoids into 2D Systems such as extracellular matrix-coated dish. The organoid-derived monolayer contains various epithelial cell lineages and enables the introduction of microorganisms via direct addition to the culture media.	1. The accessibility of the apical side of organoids is enhanced and the introduction of microorganisms can be achieved with an easily applicable setup. 2. This method can be applied to high-throughput experiments and can effectively reduce group differences caused by irrelevant variables in comparison or screening experiments. 3. Long-term co-culture with anaerobic bacteria can be achieved by incubating organoids in an aerobic environment while maintaining the apical chamber of a Transwell insert in an anaerobic environment. 4. Combined with the air-liquid interface or the microfabricated collagen scaffold array of crypt-like invaginations, this method can partially reconstruct epithelial-mesenchymal interactions and the crypt-like and villus-like structures.	1. The inoculation process caused mechanical damage to the organoids, and 2D organoids cannot reflect the structural features of lumen. 2. Certain bacterial media such as tryptone-yeast extract-glucose (TYG) and brain heart infusion may be toxic to the monolayers during introduction. 3. The success rate of establishing functional epithelial monolayers varies between different donors, which may limit its applicability. 4. Optimization measures such as providing continuous nutrient replenishment, creating an anaerobic chamber for obligate anaerobic bacteria, combining the air-liquid interface and collagen scaffold technology are costly and time-consuming.
3D organoids with growing reversed polarity^84,218,219^	Making the apical surface evert to face the media and introducing microorganisms by adding them directly to the culture medium.	1. This method simplifies the introduction of microorganisms while maintaining the 3D structure of organoids. 2. Without extracellular matrix affecting distribution, suspended apical-out organoids can be synchronously exposed to experimental agents and microorganisms. 3. Suspended organoid culture can be divided into multiple wells for different experimental conditions, which is more suitable for high-throughput experiments.	1. This method does not guarantee complete polarity reversion, so it is difficult to distinguish between the apical and basolateral interactions. 2. The mucus can be easily washed out, making it easier for foreign substances to enter the organoids. 3. Transferring apical-out organoids to new media is a time-consuming and iterative process. 4. Apical-out organoids exhibit slower proliferation and accelerated differentiation, suggesting that some of the pathways have been altered and may interfere with host-microbe interactions.
Microfluidic platforms^225–229^	Microfluidic platforms, including organ-on-a-chip, HuMiX and GuMI, are micro-engineered systems generated based on industrial computer microchips by microfabrication methods. Microfluidic platforms can provide organoids with precisely programmed biomimetic microenvironments and introduce microorganisms into organoids with ease and precision.	1. This method enables microbial diversity in organoids by tuning chemical gradients, oxygen gradients, dynamic mechanical stress and even incorporating multiple cell types and connecting multiple tissue platforms. 2. Simulation of key characteristics of the human gut and reconstruction of the mucus layer provides a better model for studying microbe-host interactions. 3. Standardized and automated organoid-on-a-chip enables high-throughput experiments. 4. Long-term coculture can be achieved through the continuous supply of nutrients and scavenging of metabolites via the microfluidic platform.	1. The complexity of organ structures and the heterogeneity of individuals determine that this method cannot fully simulate the real situation, so the applicability of this method needs to be discussed. 2. This method integrates programming, biochemistry, biomechanics, materials science and other disciplines and requires the cooperation of multiple teams and platforms, thus greatly increasing the experimental threshold and cost.

### Organoids simulating microbial interactions with the digestive organs

Several key questions to answer when studying host-microbe interactions include how microbial communities are established and maintained, patterns of microbe-host interactions and parameters that can be used to detect them, and the mechanism and targets of the microbiome contributing to disease development. Organoids cultured in growth-factor-enriched media and differentiated into various epithelial cells are emerging as a key model for deciphering pathogen invasion mechanisms due to their accurate representation of cellular heterogeneity and entry receptor expression patterns.^230,231^ Several pathogens associated with intestinal diseases have been studied in organoids. For example, *enterohemorrhagic* *Escherichia coli* (*EHEC*) can colonize a monolayer of human colonic organoids, and *EHEC* has been observed to reduce intestinal mucus, disrupt microvilli structure, and facilitate bacterial entry and infection of epithelial cells.^232^ Matrix-embedded, monolayer, or transplanted mouse organoids exposed to Shiga toxin (Stx)-producing *Escherichia coli* show increased transepithelial permeability; microinjection of the highly potent bacterial toxin Stx2a into human intestinal organoids can trigger abnormal upregulation of a variety of key structural proteins and tight junction proteins, epithelial injury, and apoptosis.^232–234^ *E. coli* pks^+^ strains are thought to cause CRC through DNA damage and chromosomal instability; after repeated microinjection of pks^+^ strains, the unique mutational signature in a subset of the human CRC genome can be detected by whole-genome sequencing in *E. coli* intervened healthy human intestinal organoids, after up to 5 months of intervention.^217^ Interestingly, following a similar intervention, another strain, *Enterotoxigenic Bacteroides fragilis* (*ETBF*), was found to promote CRC through a nongenomic mechanism. *ETBF* did not generate a distinct set of mutational signatures when incubated with human colonic organoids; instead, *ETBF*-induced tumors were driven by homologous errors in mismatch repair and recombinant DNA damage repair, suggesting that ETBF colonization is a potential threat for sporadic CRC or hereditary neoplastic diseases.^235^ *Clostridium difficile* (*C. difficile*) is an anaerobic, gram-positive, toxigenic bacterium that is a major infectious cause of nosocomial diarrhea.^236^ Microinjection of toxigenic *C. difficile* or its virulence factor TcdA into organoids resulted in loss of epithelial barrier function, marked redistribution of the Tight Junction (TJ) proteins, and downregulation of mucin 2 expression.^237,238^ *TcdB* of *C. difficile* infection of HIOs inhibits stem cell repair capacity and delays epithelial cell renewal.^249^ Infection with the major foodborne enteric pathogen *Listeria* affects goblet and paneth cell numbers, upregulates muc2 and lyz expression, and reduces the expression of genes related to the Notch pathway. Increased secretion of proinflammatory cytokines disrupts organoid morphology.^239,240^ *Enterococci* are the leading cause of multidrug-resistant infections.^241,242^ Cocultivation with *Enterococcus faecalis* family pore-forming toxins (Epxs) impairs HIO.^243^ *F. nucleatum* is a known pathogenic bacterium that mediates the pathogenesis of CRC by affecting cancer cell proliferation, exacerbating intestinal inflammation, or interfering with the immune microenvironment.^244–247^ Intervention with *F. nucleatum* stimulates the secretion of proinflammatory cytokines and the activation of multiple inflammatory signaling pathways, such as the NF-kB pathway, promoting intestinal inflammation in colonic epithelial cells of human colonoid monolayers.^248^ *Clostridioides difficile* Toxin B (TcdB) infection of HIOs inhibits stem cell repair capacity and delays epithelial cell renewal.^249^ Colonization of *Helicobacter pylori* is associated with GC, which causes most gastric ulcers by inducing hypersecretion of gastric acid.^250–252^ Gastric organoids from different sources have been used to replicate pathological conditions in vivo and to robustly mimic the gastric epithelial response to *H. pylori* infection in an in vitro system.^253–255^ *H. pylori* infection activates nuclear factor kappa B (NF-kB) to bind to the RAS protein activator-like 2 (RASAL2) promoters, inducing its expression and regulating tumorigenesis; RASAL2 silencing reduces nuclear β-catenin levels and disrupts organoid formation in gastric tumors.^256^ In a simulated acute *H. pylori* infection, an increase in inflammatory genes was rapidly observed by microinjection of *H. pylori* into the organoid lumen.^221^ It has also been implicated in immune regulation, with studies showing that *H. pylori* treatment increases PD-L1 expression in organoids; anti-PD-L1 antibodies result in *H. pylori* infection-induced organoid death when cocultured with autologous cytotoxic T lymphocytes and dendritic cells from patients.^257^ Although the most important organ affected by the intestinal microbiota is still the gastrointestinal tract, much evidence has proven that the pathological mechanism of the microbiota or its metabolites by destroying the mucosal and epithelial barrier or releasing proinflammatory factors to mediate inflammation or the immune response of other digestive organs, some of which have also been tested in organoid models.^51,258–268^

### Viruses

Organoids have also become powerful models to simulate the occurrence of digestive system diseases mediated by viral infections. Human noroviruses (HuNoVs) cause nonbacterial acute gastroenteritis in patients of all ages and are highly contagious.^269,270^ Intestinal organoids were cultured in monolayers infected with norovirus strains to monitor their replication and assess the virucidal efficacy of novel disinfectants.^269,271,272^ In addition, human digestive organoids can also be used to identify norovirus inactivating factors.^273^ Severe acute respiratory syndrome coronavirus 2 (SARS-CoV-2), the agent that causes coronavirus disease 2019 (COVID-19), has caused a global acute respiratory disease pandemic; as a highly contagious and pathogenic coronavirus, its emergence seriously threatens human health and public safety.^274–276^ The SARS-CoV-2 S-glycoprotein binds to angiotensin-converting enzyme (ACE)-II, enters the lungs via trypsin, and causes a large viral load through a rapid replication mechanism, resulting in symptoms of viral pneumonia in patients.^277,278^ Apart from lung damage caused by SARS-CoV-2, symptoms have also occurred in multiple other organs, and many research groups have used organoid models to understand cellular responses to SARS-CoV-2 and the resulting damage.^277,279,280^ Intestinal organoids infected with SARS-CoV-2 from different donors showed that the levels of viral replication in small intestine and colon-derived organoids were orders of magnitude different; virus-infected organoids showed the highest infectivity to omicron variant spike protein (S), and the susceptibility to infection was correlated with the level of ACE2.^281^ SARS-CoV-2 altered ACE2 expression in goblet cells, and efficiently tested drugs, such as remdesivir, inhibited SARS-CoV-2 infection at low molar concentrations and rescued the morphology of the organoids.^282^ Interferon-induced transmembrane (IFITM)-derived polypeptide or a target antibody inhibited SARS-CoV-2 entry and replication after SARS-CoV-2 entered gut organoids.^283^ SARS-CoV-2 can effectively infect the human liver and biliary organoids.^284–286^ The SARS-CoV-2 virus replicates rapidly in infected organoids to induce proinflammatory cytokine/chemokine production, local hepatocyte and bile duct cell damage, and consequent bile acid accumulation.^287^ SARS-CoV-2 efficiently influences the gastric epithelium, and when gastric organoids were generated from biopsies from patients of different ages, the results showed that pediatric and late-stage fetal gastric organoids were susceptible to SARS-CoV-2 infection, and viral replication was markedly reduced in undifferentiated organoids from early fetuses and adults.^219^ Hepatitis B virus (HBV) is a partially double-stranded DNA virus belonging to the family Hepatoviridae, and chronic HBV infection can progress to end-stage liver diseases such as cirrhosis and HCC.^288,289^ Human liver organoids are a useful platform for mimicking HBV infection and related tumorigenesis because they retain the structure and gene expression patterns of human hepatocytes.^290–294^ HBV infection of organoids replicates the viral life cycle and virus-induced liver dysfunction.^295^ Organoids infected with HBV in vitro can also produce covalently closed circular DNA (cccDNA) and HBV early antigen (HBeAg), express HBV RNA and protein intracellularly, and generate infectious HBV.^290^ iPSC-derived liver organoids (IPSC-LOs) are more susceptible, and HBV infection not only supports HBV transmission but also downregulates hepatic gene expression, induces the release of early markers of acute liver failure (ALT/LDH), alters liver ultrastructure, and leads to hepatic dysfunction in organoids.^296^ EBV is etiologically associated with at least 8% of GC cases (designated EBVAGC), including distinct genetic and epigenetic GC subsets.^297,298^ In paired normal and cancer-derived organoids from the same patient, EBV infects only the cancer organoids.^299^ Organoid models have demonstrated their value in validating the safety and efficacy of antiviral treatment.^272,300–302^ These in vitro models are able to leverage prior knowledge of viral biology.^293^ Finally, the integration of genetically engineered organoids or biopsy-derived organoids to mimic specific and rare subtypes of digestive diseases could help identify effective treatments for a small subset of patients with rare diseases from whom organoids are established.^303–308^

## Digestive system organoid models and precision and personalized therapy

In the era of precision therapy, organoid models can be used to accurately predict individual responses to treatments.^309–312^ The global application of organoid technology has led to unprecedented progress in many diseases; given the heterogeneous composition of tumors, no single treatment approach is suitable for all patients, and organoids derived from tumors are rapidly becoming a vital tool for the individual selection of treatments.^313–316^ Organoids can be used to study mutations and treatment options at different disease stages.^317,318^ In addition, organoids can be generated by performing multiple rounds of biopsies of patients at different time points to continuously assess their response to treatment, detect emerging resistance, and subsequently screen new treatments.^51^ These patient-derived organoids, or organoids that mimic disease, can be tested for medicine selection, cell therapy, immunotherapy, gene correction, or combinations of several treatments for individuals.^312,313^

### Personalized medicine therapy

Personalized therapy is treatments performed for each patient and using a personalized disease model with similar characteristics to the primary tumor will undoubtedly give a more accurate prediction of the patient’s response to a given treatment.^161,319,320^ Patient-derived organoids retain donor-specific properties and disease-associated differences.^172,321–323^ The selection of drug therapy using organoid models can be divided into two aspects: selection of existing drugs (including the reuse and repositioning of old drugs) and screening of new drugs/chemicals. The latter can be classified as drug discovery.

#### Drug screening to delineate treatment strategies

AdSC-based organoid technology enables drug responsiveness testing in a fraction of the time needed for previous methods.^324^ Additionally, no single treatment is effective for all patients, suggesting that PDOs need to be tested to determine the best treatment regimen for the individual patient.^320^ Whether the levels and responses of organoids in many drugs and chemoradiotherapy are comparable to in vivo therapy has been extensively examined from multiple perspectives. Some studies showed that the chemoradiotherapy response of rectal cancer organoids was very well correlated with patients, with a specificity of 91.97%, an accuracy of 84.43%, and a sensitivity of 78.01%.^129^ Endoscopic biopsy of esophageal cancer generates PDO and compares clinical outcomes after neoadjuvant therapy with in vitro PDO responses.^325^ Organoids from metastatic CRC and GC/EAC PDOs have been used for mid-range drug screening, and parallel drug studies in organoid xenografts confirm in vitro and in vivo therapeutic response correlations.^324^ The response of GC PDOs to two clinically used paclitaxel (PTX) nanoformulations, albumin-bound PTX (Albu-PTX) and liposomal PTX (Lipo-PTX), was comparatively assessed using a GC PDO model that reproduced the therapeutic superiority of Lipo-PTX over Albu-PTX.^151^ The clinical response to drugs can be predicted from patient-derived data, which can be used to create personalized treatment strategies based on the specific response of organoids to existing treatments.^326^ Organoids from patients with metastatic gastrointestinal cancer receiving commonly used treatments are used to predict treatment response and tumor differences between patients and to model intra-patient tumor response heterogeneity between different chemotherapeutics.^324^ By obtaining PDO during a diagnostic biopsy, multiple regimens can also be tested simultaneously and applied at clinically relevant intervals.^327^ For individualized treatment, the most valuable direction for research is how to obtain tissue through biopsy and other means before surgery and to test radiotherapy and chemotherapy treatment as quickly as possible to guide the selection of an appropriate radiotherapy dose, chemotherapy drugs, and adjuvant therapy. Researchers have optimized intraoperative drug screening with 96-well plate-cultured organoids and made the provision of molecular typing, drug screening, and individualized adjuvant therapy guidance for each patient within one week become feasible.^328^ Newer techniques can be used to generate GC organoids in endoscopic biopsies of patients with advanced metastatic GC who are not suitable for surgery and then test the efficacy of multiple standard drug regimens and combinations in a relatively short period of time, providing valuable prognostic and therapeutic options for this type of patient.^329^ Additionally, if the dose-response curves of PDOs can be established to reproduce in vivo responses, biopsies can be processed immediately to produce organoids for high-throughput drug screening assays, simulating therapeutic responses to various conventional and investigational treatments for PDAC changes, as well as differences in individual patient responses to overall treatment.^330^ Organoid biobanks can be used to detect resistance to existing drugs and select alternative treatment options. Hedgehog pathway inhibitors combined with 5-fluorouracil (5-FU), or irinotecan have potent antitumor effects on drug-resistant colorectal cancer in organoids.^331^ A drug screening test was conducted for the treatment of oxaliplatin-resistant CRC by PDOs, and the results showed that inhibition of KLF5 by ML264 was mainly mediated by antiapoptotic effects and restored oxaliplatin sensitivity in CRC.^332^ Response analysis of PDAC organoids to five commonly used chemotherapeutics (5-FU, irinotecan, oxaliplatin, gemcitabine and paclitaxel) showed that one-third of PDOs were resistant to all five drugs, and half of the patients who provided these PDOs were highly sensitive to targeted drugs.^172^ The multicellular HCC organoid (MCHO) contains a large number of stromal cells that have some influence on drug penetration, and interference with YAP/TAZ transcriptional activity in hepatoma cells significantly increases the penetration of verteporfin into MCHO.^333^ Organoid technology has been shown to play a key role in gene-drug association theory in individualized and targeted therapy. For example, TP53-mutated PDOs are resistant to Nutlin-3a, an MDM2 inhibitor, KRAS-mutated organoids are extremely resistant to inhibitors of ERBB, and RNF43-mutated colon tumor organoids are dramatically susceptible to Wnt secretion inhibitors.^334^ ERBB2-amplified PDOs respond well to lapatinib, a dual ERBB2/EGFR inhibitor.^335^ Trametinib combined with celecoxib is the most effective strategy for KRAS- and TP53-mutated advanced colorectal cancer.^319^ Colorectal cancer organoids with RAS mutations may respond to combination therapy with MEK and pan-HER inhibitors.^336^ As in vitro models for drug safety evaluation, small and large intestine organoids have been used in toxicology studies to predict drug-induced gastrointestinal toxicity, such as assessing transcriptomic responses associated with viability and apoptosis following exposure to doxorubicin and gefitinib as a physiological endpoint.^337,338^ Human intrahepatic cholangiocyte organoids (ICOs) can recapitulate necroptosis associated with bile duct disease, providing a useful in vitro platform for biliary cytotoxicity studies and preclinical drug evaluation.^191^ The applicability of hepatic organoids derived from human iPSCs for drug toxicity assessment was demonstrated by comprehensive functional analysis of CYP450-mediated drug metabolism.^339^ These cases demonstrate the versatility of organoids and their consistency with in vivo therapy, and these validation results promote the ubiquitous role of organoids in drug selection and prediction for clinical treatment.

#### Drug discovery

Organoids that replicate the characteristics of in situ tissue can also be used for more accurate drug discovery models. In this approach, drugs or compounds are tested on organoids of different disease origins. These drugs or compounds include agents in preclinical testing, agents in clinical development, and agents clinically used for the other diseases at hand. Currently, there are many examples of drug discovery using organoids in preclinical or clinical practice.^91,340^ For drug discovery, the size of the organoid library and the size of the drug or compound library should be selected appropriately. While some drug-response models are available for processing large compound libraries, others are suitable for screening some disease types that are sensitive to single chemotherapeutic drugs or combinations of targeted drugs. In other words, some effective targeted therapeutic drugs or compounds can be identified by drug screening with different throughputs in PDOs. Clevers’ laboratory described the first mid-scale drug screen using its CRC organoid biobank; the organoid is plated and processed with an extensive library of cancer-targeting compounds.^323^ Huch’s laboratory initially conducted a mid-scale drug screen of 29 anticancer compounds, including those in clinical use or in development, and LC PDOs showed variable sensitivity to some of the compounds.^194^ Another team assessed the feasibility of large-scale drug screening using PDAC organoids by exposing PDAC PDOs to 76 targeted therapy drugs and classical chemotherapeutics.^173^ Some drugs in clinical development or preclinical small molecules have been identified as target drugs for a certain disease and may also be found to be suitable for the treatment of other diseases in the experiment. Organoids are used to test drugs or compounds that previously targeted other diseases, meaning that old drugs are used for new diseases; this approach eliminates many pharmacokinetic and organic toxicity tests and allows for faster clinical applications.^341–343^ Of course, it should be noted that it is not limited to the drugs in past and present clinical use for the purpose. The FDA-approved chronic myeloid leukemia (CML) drug omacetaxine has been shown to be the most potent small molecule in HCC PDOs.^344^ Through PDAC PDO screening, CHK1 inhibitors for breast and ovarian cancer (ongoing trials: Phase I or Phase II) are also effective in pancreatic cancer.^171^ PCSK9 is a therapeutic target for hypercholesterolemia and dyslipidemia but is also emerging as a potential target for colorectal cancer, as PCSK9 inhibitors inhibit the growth of APC/KRAS mutant CRC organoids.^199^ Organoid models are also a promising tool for small-molecule screening in some intractable diseases and tumor metastases to discover potential novel therapeutic agents. Fangchinoline has been shown to be a potent small molecule that can dose-dependently inhibit the growth of patient-derived organoids by directly targeting NOX4, thereby reducing non-small cell lung cancer (NSCLC) metastasis.^345^ The VprBP inhibitor B32B3 is able to reduce colonic organoid growth by blocking H2AAT120p and activating the normal transcriptional program.^346^ Human pancreatic islet organoids respond to the HIF-1α inhibitor PX-478, and long-term exposure to high glucose increases the glucose-induced insulin secretion stimulation index, suggesting that the HIF-1α inhibitor PX-478 has the potential to act as an antidiabetic agent.^347^ AT-rich interacting domain 1 A (ARID1A), an important subunit of the chromatin remodeling complex, carries a heterozygous mutation in most human GC cases, and tumor organoids with ARID1A heterozygosity show growth inhibition by combination therapy with TP06 and Nutlin-3, an epigenetic inhibitor and a p53 agonist, respectively, offering a new option for GC therapy.^348^ Investigating combination drug therapies in organoids is a good strategy to combat drug resistance and emerging diseases in the digestive system.

### Stem cells and regeneration

Adult stem cells are the cornerstone of the renewal of multiple cell types in multiple tissues.^48,349^ To unravel the regulatory mechanisms by which stem cells act as key determinants of regeneration following tissue trauma or inflammatory injury, extensive research efforts have been conducted to elucidate the plasticity of tissue stem cells.^350–355^ Stem cell-derived organoids have unparalleled advantages in the field of regeneration because they contain not only stem cells but also their differentiated progeny and can be continuously cultured and stored; they can recapitulate the regenerative capacity of the epithelium and restore homeostasis after damage.^98,356,357^ Numerous studies have shown that organoid technology has the potential to provide alternative organ replacement strategies for various types of digestive diseases.^41,358^ Transplanting gut organoids into mice is also the best model to study stem cell fate determination and microenvironmental interactions, and the transplantation of normal human gut organoids onto the mouse gut surface enabled the prioritized maintenance of stem cells to reproduce human intestinal epithelial tissue in a heterograft system.^65^ After organoid transplantation in the Rag2/DSS colitis model, donor cells achieved “mucosal healing” and rescued the pathology of DSS colitis.^351,359^ Extensive resection of the small intestine can lead to malabsorption and weight loss, a condition called short bowel syndrome (SBS). In a rat model of SBS, ileal organoid transplantation yields a well-functioning small intestine and significantly mitigates intestinal failure.^360^ Liver transplantation is a treatment method to restore liver function in patients with irreversible liver failure.^361,362^ Liver organoids, similar to liver lobules, are expected to be used in regenerative medicine to generate a source of cells to overcome the current shortage of transplant organs.^363–365^ Liver organoids generated from expanded hepatic progenitor cells (HPCs) reconstituted hepatic interstitial structures after implantation in allogeneic mice.^366^ Cholangiocellular organoids can be used for the repair of human bile ducts.^367^ AdSCs have a remarkable ability to generate organoids, which also help identify signals that control the lineage fate of stem cell progeny and guide models of tissue plasticity during injury and regeneration.^350,368^ Thus, in addition to direct organoid transplantation, understanding the mechanism of stem cell plasticity for endogenous regeneration regulation is also a promising therapeutic direction.^167,369–371^ It is generally believed that dedifferentiation of epithelial cells to stem cells and the existence of a reserve stem cell pool are two sources of stem cell-driven regeneration.^372,373^ The reserve stem cell theory posits that repair and regeneration depend on the re-initiation of early transcriptional developmental programs in quiescent, viable reserve stem cell populations under homeostatic conditions. For example, p57^+^ cells exhibit quiescent stem cell activity, undergoing a dynamic reprogramming process of differentiation as part of constitutively activated spatiotemporal reprogramming and as facultative stem cells, supporting regeneration after injury.^374^ Single-cell sequencing of highly proliferating intestinal organoids obtained using a combination of chemicals and factors revealed variable expression of regenerative stem cells, such as in vivo damage-responsive Clu^+^ revival stem cells or Lgr5^+^ stem cells for tissue repair through epigenetic reprogramming.^350^ The theory of dedifferentiation suggests that epithelial cells are uniquely plastic, enabling them to dedifferentiate and replenish the pool of cycling cells lost upon damage; thus, by reverting to a more primitive state, the organ allows itself to remodel tissue patterns into homeostatic tissue compartments and induce regeneration.^375–377^ For example, in the absence of Lgr5^+^ stem cells, intestinal cell lines expressing intestinal alkaline phosphatase (Alpi^+^) dedifferentiate and further distribute and localize to the bottom of the crypt, becoming Lgr5^+^ stem cells.^378^ In vitro dedifferentiation of Alpi^+^ intestinal epithelial cells into lgr5^+^ stem cells could be reproduced by intestinal organoid models.^378^ Any theoretical knowledge of the emergence of stem cells that regulate injury repair in the context of injury and the molecular or signaling pathways that can be used to regulate it is beneficial for regeneration. Some factors or signaling pathways have been identified to drive the process of cell type differentiation as well as tissue regeneration.^48,379,380^ The Yes-associated protein 1 (YAP) and Wnt signaling pathways are involved in guiding regeneration and homeostatic tissue renewal.^381^ YAP is normally localized only in the nucleus in cryptic basal stem cells but becomes a nucleus in most intestinal epithelial cells in a kinase dependent Src family manner during intestinal regeneration or organoid growth after irradiation.^382^ CREPT is expressed in intestinal crypt Lgr5^+^ ISCs and maintains steady state regulatory factors; in the process of intestinal regeneration, it activates the Wnt signaling pathway.^383^ The use of interstitial stem cell-conditioned culture medium, including paracrine factors and the Wnt/Notch signaling pathway, was able to partially restore radiant organoids.^384^ These new insights into the regulation and fate determination of stem cells in organoid facilitate the development of therapies that harness regenerative capabilities to treat digestive disorders.^385^ Activation of endogenous stem cells is a complex process that requires the identification of various factors that affect the balance of stem cell proliferation and differentiation and the formation of finely regulated networks to control endogenous stem cell activation and promote regeneration.^386^ These organoids also provide valuable mechanistic insight into the development of stem cells and their niches while monitoring the development of these cells into mature functional lineages by regulating various signaling pathways, including Wnt, BMP, EGF, Notch, and FGF. Small molecule or nutritional factor manipulation of specific lineages can use organoid platforms to provide proof of concept and a deeper understanding of organ lineage specification; it can even identify potential intervention drugs.^387–389^ Epidermal growth factor is an important fate determinant that differentiates the surface and interior of human gastric glands and binds to BMP signaling to control pit cell differentiation from parietal or primary cells.^390^ The small molecule isoxazole 9 (ISX-9), a neurogenic modulator, enhances secretory progenitor cells in organoids by upregulating the transcription factor Pax4, a component of early human endocrine-specific progenitors.^391^ The serotonin receptor agonist Bimu8 could increase the density of L-cells in primary human colonic organoids.^392^ Pretreatment with L-arginine induces the stemness of the stem cell pool and preserves the gut response to TNF-α and 5-fluorouracil.^393^ Nuclear exportin 1 inhibitor regulates intestinal stem cell fate independently of known differentiation cues and significantly increases paneth cell abundance in organoids.^394^ Bile acids (BAs) and G protein-coupled bile acid receptor 1 (GPBAR1, also called TGR5) expression on gut stem cells regulates epithelial self-renewal and fate determination, cocultures of gut stem cells with BAs and TGR5 agonists improved gut organoid growth, and YAP1 and SRC inhibitors blocked TRC5-activated organoid growth.^395^ Utilizing multiple layers of genetic intervention on organoids platform is another approach to uncover the origin of various cell fates and determine the underlying signaling factors in the digestive system.^396^ Organoids with genetic knockout of protein phosphatase Mg_2_^+^/Mn_2_^+^‑dependent 1A (PPM1A) enhanced YAP/TAZ retention in the cytoplasm, leading to reduced cell proliferation and downregulation of RALY inhibition of development in an organoid model.^397^ CRISPR-Cas9-engineered human iPSCs with truncated HNF1α^p291FsinSCs^ grown as 3D pancreatic organoids were shown to abolish HNF1β function and reduce pancreatic progenitor and β-cell differentiation.^398^ Angiotensin converting enzyme 2 (ACE2)-specific knockout gut organoids show decreased Lgr5 and Ki67 levels.^399^ Endoplasmic reticulum membrane protein complex subunit 3 (Emc3) is a determinant of the maintenance of gut mucous homeostasis, and the knockout of Emc3 led to enhanced endoplasmic reticulum (ER) pressure and destroyed Paneth cell function in the stem cell niche, leading to the failure of the culture of gut organoids.^400^ In addition to detecting the regenerative process in response to two kinds of regenerative stem cell patterns, organoid models can also detect how other cell types affect stem cell-mediated regeneration. As an integral part of the intestinal stem cell tissue microenvironment, Paneth cells not only form the boundary between the stem cell niche and epithelial organoid precursors but also provide essential Wnt3 for Lgr5^+^ stem cells.^401,402^ Immune cells, such as intraepithelial lymphocytes (IELs), also play an important role in maintaining homeostasis by secreting factors that affect the balance of stem cell self-renewal and differentiation.^403^ High-purity stem cells can be obtained from organoid culture; thus, new stem cell marker genes may be discovered, or the roles of cycling and quiescent stem cells may be investigated in lineage selection and differentiation; for example, CD24, EphB2, Krt17 and CD166^+^/GRP have been identified as novel stem cell markers using organoid cultures.^355,404–406^ Organoid models largely reproduce these processes and mechanisms, and further access to organoid systems can be used to identify novel physiologically relevant targets, in this case using multivariate phenotypic screens to elucidate the process of gut regeneration.

### Immunotherapy

Tumor-induced immunosuppression is an important reason for evading immune surveillance attack.^407^ Immune checkpoint therapy (ICT) strategies targeting PD-1 and CTLA-4 can reverse tumor immunosuppression to a certain extent and achieve better therapeutic effects, but the clinical response is still low. It is necessary to further elucidate the mechanism of tumor immunosuppression and find new immunotherapy targets and strategies.^408^ The role of organoids in immunotherapy can be summarized in four directions: (1) reconstruction of the tumor immune microenvironment by coculture of organoids and immune cells as a platform for exploring and evaluating immunotherapy strategies;^223^ (2) exploration of the response of different phenotypes of cancer to immunotherapy and factors affecting the efficiency of immunotherapy by manipulating the gene expression of tumor organoids or changing the culture environment of tumor organoids in combination with gene editing technology;^409–412^ (3) comparison of different immune (adjuvant) therapies using tumor organoid tests to provide optimization ideas;^411,413,414^ and (4) the use of organoids (or cultures) to induce the generation of functional T cells as biological factories for the production of immunotherapeutic drugs.^415–418^ Digestive tumor organoids derived from tumor tissue or PSCs have potential applications in personalized immunotherapy.^312,419,420^ These organoids are cocultured with immune cells to construct or maintain complex tumor tissue microenvironment structures, which can be used for immunotherapy research for digestive system diseases and potential therapeutic development for personalized therapy.^419,421,422^ Organoid models of immunotherapy are slowly being built to support immune cell survival and study interactions between T cells and tumor cells.^415,418^ The type and source of immune cells included in the coculture can be determined according to the research scenario, for example, autologous/exogenous expanded peripheral blood mononuclear cells or specific activated immune cells derived from tumor biopsies or surgically resected human colon cancer samples.^415^ In a coculture of pancreatic tumor organoids with peripheral blood lymphocytes, tumor-dependent lymphocyte infiltration was observed in these models.^423^ Likewise, CRC and NSCLC organoids were cocultured with autologous peripheral blood mononuclear cells (PBMCs), cytotoxic T lymphocytes (CTLs) were successfully expanded, and tumoricidal effects were observed.^418^ Another study turned to the use of exogenous immune cells cocultured with colon cancer spheroids/organoids, and allogeneic T cells and NK cells were also able to rapidly infiltrate tumor spheroids, leading to immune-mediated tumor cell apoptosis.^424^ Through the successful activation of immune cells by coculture with tumor-tissue-derived organoids, one can not only observe the killing effect of immune cytotoxicity on malignant cells in vitro but also functionally test the efficacy of immunotherapy in specific patients, which is helpful to understanding potential biological principles. In an organoid model of human steatohepatitis, CD47 blockade prevented hepatic stellate cell activation and progression of steatohepatitis.^425^ T-cell depletion plays an important role in tumor immune escape. Identifying potential compounds that target T-cell depletion and enhance immune checkpoint inhibitor (ICI) responses to suppress immune evasion is a viable strategy. For example, dexamethasone inhibits immune evasion by decreasing PD-L1 expression and Indoleamine 2,3-dioxygenase 1 (IDO1) signaling.^426^ PDO models combined with gene editing techniques are used to determine which factors mediate disruption of cell-autonomous mechanisms that protect tumors from immune clearance, thereby improving the efficacy of immunotherapy. Human pancreatic cancer organoids with mutations in RNF3 are sensitive to the killing effect of CTL.^427^ In addition to testing the interaction and killing function between immune cells and tumor organoids, another dimension of research can be added, such as observing the impact of external stimuli on the immune environment and the effects of immunotherapy. Furthermore, these systems also showed excellent performance in examining the efficacy of combination therapies. Immune checkpoint blockade (ICB) therapies, such as PDL-1 antibodies, face the dilemma of low efficacy, and clinical and preclinical experiments have begun to test the efficacy of combination therapies. As a combination therapy, PD-1 blockers in combination with TBK1/IKK inhibitors can counteract resistance and improve the response to PD-1 blockers by helping to overcome the immunosuppressive microenvironment of immune systems.^428^ CDK4/6 inhibitor combined with anti-PD-1 blocking antibody treatment of tumor spheres increases the number of infiltrating T cells and enhances antitumor activity.^429^ Chimeric antigen receptor T-cell therapy (CAR-T), a new type of cellular immunotherapy, has shown high efficiency and wide clinical application value in the treatment of various hematological malignancies in recent years.^430^ This therapy generates and expands a patient’s autoimmune T cells in vitro, allowing them to more efficiently recognize specific antigens expressed by tumor cells. When reinjected into patients, these CAR-T cells can efficiently and specifically recognize and attack tumors.^431^ Organoids have also been shown to be effective platforms for evaluating CAR-T-cell potency and tumor specificity.^417,432,433^ In HER2-expressing colorectal cancer organoids obtained from patient biopsies, the addition of anti-HER2 CAR-T cells alone resulted in minimal killing, while the combination of CAR-T cells with the apoptosis antagonist birinapant induced powerful lethality of organoids.^434^ Colonic organoids were also used to track CAR-mediated NK-cell cytotoxicity.^127^ Recent studies have shown that it is possible to perform iPSC-derived T cells. These stem cell-derived T cells display full immunity and high tumor killing similar to human peripheral blood TCRαβ T cells. This novel CAR-derived T-cell circumvention of donor-derived defects will greatly promote the promotion of CAR-T therapy.^435^ With the continuous maturation and application of the coculture system of immune cells and organoids, it is believed that the organoid model will be a major asset to the field of immunotherapy.

### Other treatments

Organoids can serve as a good platform for evaluating gene therapy, in which gene editing technologies are used to repair genetic deficiencies in patients.^311,436,437^ For example, the use of CRISPR gene editing technology to correct CFTR mutations in intestinal organoids of CF patients has shown that gene repair can correct the CFTR locus in patient-derived CF organoids and restore the normal response of organoids to forskolin treatment.^438^ Organoids can also be used as an alternative test model for FMT therapeutic strategies. Given the compelling evidence that many human diseases are associated with microbiota dysbiosis, restoring physiological microbiota balance and composition may improve disease symptoms.^439–443^ FMT has been tested in a variety of diseases, such as malignancies, metabolic syndrome, neurological diseases, and autoimmune diseases, and has shown surprising therapeutic effects.^444–449^ An important concept in this framework is the need to identify well-defined beneficial traits in the microbiome and validate the impact of beneficial bacterial combinations on individuals.^450,451^ Organoids would be good models to test how to determine the role of target microbiota and the effect of target microbiota.

### Digestive organoid biobanks and clinical studies

Because various organoids can be easily obtained from postoperative or biopsy tissue and cultured indefinitely, many patient-derived organoids form the basis for the creation of living biobanks.^323,452,453^ These biobanks contain many organoids from different clinical stages and histological subtypes, determined by gene expression signatures, and culture methods are regulated and optimized according to the needs of niche factors.^152,454^ Human organoids can be obtained from postoperative or preoperative biopsies and contribute to understanding disease mechanisms, drug screening to predict treatment outcomes, regenerative medicine, and developing treatment options for personalized treatment regimens.^455^ To continuously optimize clinical treatment strategies and explore the pathogenesis of diseases, organoid biobanks for various diseases have been established in recent years, among which gene mutation-related diseases and malignant tumors are the most common.^303,323,324,456^ For example, organoid biobanks derived from patients with digestive system diseases have been established; the resources in these biobanks include intestine,^57,319,324,415,457^ stomach,^61,151^ pancreas^171,174,458^ and liver organoids.^194,459^ Other more extensive organoid biobanks have been established by academic institutions (HUB, https://huborganoids.nl/) and commercial entities (American Type Culture Collection (ATCC, www.attc.org), Sigma-Aldrich, DefiniGEN (https://www.definigen.com/products/intestinal/organoid/) and Cellesce (https://cellesce.com)). These human organoids bring together a large number of adult stem cell organoids derived from primitive human tissue or organoids derived from iPSCs, providing researchers with opportunities for biomedical research and drug testing.^453^ Another benefit of building an organoid biobank is that, by combining large samples and clinical data, it is possible to retrospectively study the response of patient samples to existing drugs. In the case of ineffectiveness, mechanistic studies and drug screening can be performed to address these causes. Given these advantages, PDO can serve as an important tool for drug screening and prognostic assessment in the clinic, demonstrating their great potential in personalized and precise treatment.

Organoid-based clinical research is another emerging hotspot, including the construction of organoid biobanks, drug screening, prognostic assessment, and disease mechanism research. Organoids can be stored for a longer period and are easier to transport than primary tissues; therefore, they are gradually becoming a new favorite for large-scale cohort studies.^460^ Registered clinical trials are collecting and constructing organoid biobanks for diseases such as colorectal cancer, hepatocellular carcinoma, cholangiocarcinoma, pancreatic cancer, inflammatory bowel disease and primary sclerosing cholangitis, promoting the widespread adoption of organoid construction in the clinical diagnosis and treatment process and the standardization of the construction process (Table 2). Organoids as a platform for drug screening can improve the screening efficiency and save experimental costs. The completed and ongoing clinical trials using organoids for drug screening are mainly focused on antitumor drugs. Target diseases include pancreatic, colorectal, gastric, and biliary tract cancer, and the experimental design includes exploring the response of tumor organoids to single drugs or different drug combinations, the response of specific mutation types to drugs, and exploring the consistency between treatment responses in organoids and clinical outcomes of chemotherapy. The evaluation indicators include drug effectiveness, drug tolerance, and drug side effects. Additionally, organoids derived from genetic diseases such as cystic fibrosis can also be screened for drugs.^461^ In addition to drug screening, organoids can be used for a wider range of preclinical assessments, including response to radiation therapy, comparison of primary and metastatic cancers, biomarker screening, and assessment of prognosis and risk of recurrence. Through prior experiments on organoids, clinicians can make evidence-based decisions to reduce unnecessary harm to patients caused by decision errors and outcome uncertainty and improve the efficiency and safety of clinical care. At present, many diseases, such as allergies and irritable bowel syndrome, lack suitable disease models^462^ and the emergence of organoids provides new research ideas. By constructing organoids to simulate the intestinal environment, researchers can explore the interaction between the intestinal system and the outside factors, explore the pathophysiological mechanisms and formulate treatment strategies. Relevant clinical trials include studies of microbe-host interactions, mechanisms of allergy and intestinal disorders, the characterization of intestinal stem cells in patients, the biology of innervated sensory epithelial cells, the physiopathology of necrotizing enterocolitis, and gut inflammation in the pathogenesis of hypertension. Overall, organoid-based clinical trials continue to increase in variety and number. In addition to the abovementioned four applications, organoid-based immunotherapy^463^ and tissue regeneration research^367^ have also made breakthroughs, and corresponding clinical trials and translational results are also expected to appear in the future.

**Table 2:** Summary of organoid-based clinical trails

Application	Condition or disease	Research title	ClinicalTrials.gov Identifier
Drug Screening	Pancreatic Cancer	A Prospective, Randomized, Controlled Trial of Chemotherapy for Advanced Pancreatic Cancer Based on Organoid Drug Sensitivity Test	NCT04931381
		Guidance of Standard of Care Treatment for Metastatic Pancreatic Cancer by Drug Screening in Patient-derived Organoids: A Single Centre, Open-label, Single Arm, Phase II Trial With Feasibility Endpoint	NCT05351983
		A Prospective, Randomized, Controlled Trial of Adjuvant Chemotherapy for Pancreatic Cancer Based on Organoid Drug Sensitivity Test	NCT04931394
		Drug Screening of Pancreatic Cancer Organoids Developed From EUS-FNA Guided Biopsy Tissues	NCT03544255
		Pharmacotyping of Patient-derived Pancreatic Cancer Organoids from Endoscopic Ultrasound-guided Biopsy as a Tool for Predicting Oncological Response	NCT05196334
	Colorectal Cancer	Prospective Observation on the Accuracy of in Vitro Screening of Colorectal Cancer Chemotherapy Drugs Based on Organoids-on-a-chip	NCT04996355
		Patient-derived Organoids of RAS/RAF Wild-type Metastatic Right Colon Cancer to Test the Sensitivity and Clinical Consistency of Combined Treatment of Cetuximab.	NCT04906733
		A Prospective Multicenter Randomized Controlled Trial of the Clinical Efficacy of Neoadjuvant Therapy Based on Organoids Drug Sensitivity Versus Empirical Neoadjuvant Therapy in the Treatment of Advanced Rectal Cancer	NCT05352165
		The Culture of Advanced/Recurrent/Metastatic Colorectal Cancer Organoids and Drug Screening	NCT05304741
		Pilot Study for Ex Vivo Tailoring of Treatment in Colorectal Cancer	NCT05401318
	Gastric Cancer	The Clinical Efficacy of Patient-derived Organoid-based Drug Sensitive Neoadjuvant Chemotherapy Versus Traditional Neoadjuvant Chemotherapy in Advanced Gastric Cancer: A Prospective Multi-center Randomized Controlled Study	NCT05351398
		Consistency Between Treatment Responses in Patient-Derived Organoid (PDO) Models and Clinical Outcomes of Neoadjuvant Therapy, Conversion Therapy and Palliative Therapy in Gastric Cancer	NCT05203549
		A Prospective Observational Study on the Potential Benefit of Neoadjuvant Therapy for Advanced Gastric Cancer Based on Organoid Drug Susceptibility Screening	NCT05442138
		Q-GAIN (Using Qpop to Predict Treatment for GAstroIntestinal caNcer)	NCT04611035
	Cystic Fibrosis	Response to CFTR-modulators in Intestinal Organoids of Patients with CF Having at Least One R334W Mutation	NCT04254705
	Biliary Tract Cancer	A Prospective Feasibility Study of Multi-Platform Profiling Using Biospecimens From Patients With Resected Biliary Tract Cancer	NCT04561453
Preclinical Evaluation	Pancreatic Cancer	Establishing Organoids from Metastatic Pancreatic Cancer Patients, the OPT-I Study	NCT03500068
		Development of a Prediction Platform for Neoadjuvant Treatment and Prognosis in Pancreatic Cancer Using Ex Vivo Analysis of Organoid Culture	NCT04777604
		Development of a Prediction Platform for Adjuvant Treatment and Prognosis in Pancreatic Cancer Using Ex Vivo Analysis of Organoid Culture	NCT04736043
	Colorectal Cancer	Validation of Organoids Potential Use as a Companion Diagnostic in Predicting Neoadjuvant Chemoradiation Sensitivity in Locally Advanced Rectal Cancer	NCT03577808
		Systemic Neoadjuvant and Adjuvant Control by Precision Medicine in Rectal Cancer (SYNCOPE) - Approach on High-risk Group to Reduce Metastases	NCT04842006
	Radiation Enteritis, Inflammatory Bowel Diseases	Preclinical Evaluation of Multimodal Therapeutic Strategies in Intestinal Irradiation and Inflammatory Bowel Disease from Organoids	NCT05425901
	Esophageal Cancer	Chemoradioresistance in Prospectively Isolated Cancer Stem Cells in Esophageal Cancer-Organoid: RARE STEM-Organoid	NCT03283527
	Esophagogastric Carcinoma	Molecular Outcome Prediction of Neoadjuvant Systemic Treatment in Esophagogastric Carcinoma	NCT03429816
	Colorectal Cancer	The Exploratory Study of Patient-derived Organoids for the Prediction and Evaluation of Clinical Efficiency Effect of Colorectal Cancer Liver Metastasis	NCT05183425
		Validation of the Three-dimensional Bioprinted Tumor Models as a Predictive Method of the Response to Chemotherapy for Colorectal Cancer With or Without Liver Metastases	NCT04755907
Living biobanks	Inflammatory Bowel Disease	Prospective, monocentric cohort aiming at generating 3D organoids from human digestive samples	NCT05294107
	Colorectal Cancer	Feasibility of Establishing Patient-Derived Organoids for Rectal Cancer: A Biospecimen Collection Protocol	NCT04371198
		Tumor Immune Microenvironment Involvement in Colorectal Cancer Chemoresistance Mechanisms: a Patient-derived Tumoroids Prospective Collection From Systemic Treatment Naive Tumors	NCT05038358
	Colorectal Cancer Metastases and Hepatocellular Carcinomas	Next Generation “ of Liver Derived-organoïd Biobank: Case of Colorectal Cancer Metastases and Hepatocellular Carcinomas	NCT05384184
	Liver, Biliary and Pancreatic Cancer	A Study Designed to Develop in Vitro Models of Liver, Biliary and Pancreatic Cancer for the Investigation of Tumour Biology and Potential Therapies	NCT02436564
	Primary Sclerosing Cholangitis	Characterization of Biliary Cell-derived Organoids From Bile of PSC and Non-PSC Patients	NCT04753996
	Pancreatic Cancer	EUS-guided Biopsy of Pancreatic Mass Lesions for Developing Patient -Derived Cancer Models	NCT03140592
Mechanism exploration	Host-microbiota Interactions	Establishment of Human Organoid Lines as a Tool to Dissect Molecular Pathways of Host-microbiota Interactions	NCT05323357
	Allergy and Gastrointestinal Disorders	Establishment of Small Intestinal Human Organoids to Check the Influence of Nutrient Antigens or Therapeutic Agents	NCT03256266
		Improving the Diagnosis of Food Allergy and Food Intolerance by Determining Mucosal IgE and Inflammatory Markers and Validating With Intestinal in Vitro Organoids	NCT05259826
		Bestimmung Des Einflusses Von Nahrungsmitteln Auf Die Darmschleimhaut Mit Der Konfokalen Laserendomikroskopie Und Humanen in Vitro Organoiden	NCT05056610
	Intestinal Stem Cells Characterization	Intestinal Stem Cells Characterization in Intestinal Organoid Culture from Inflammatory Bowel Disease and Intestinal Polyposis Patients	NCT02874365
	Specimen acquisition	Evaluation and Comparison of the Growth Rate of Pancreatic Cancer Patient-derived Organoids Generated from Matched Fine Needle Aspirations (FNA) and Fine Needle Biopsies (FNB)	NCT03990675
	The biology of innervated sensory epithelial cells	The Innervation of Human Gut Sensory Epithelial Cells	NCT02888587
	The physiopathology of Necrotizing Enterocolitis	Development and Use of a Tissue and Human Enteroid Biorepository to Study the Pathophysiology of Neonatal Necrotizing Enterocolitis	NCT04549727
	The gut epithelium properties in hypertension	Gut Inflammation and Gut-Gut Microbiome Interactions in the Pathogenesis of Hypertension	NCT04497727

## Current limitations and advantages of organoids

**Fig. 4:**
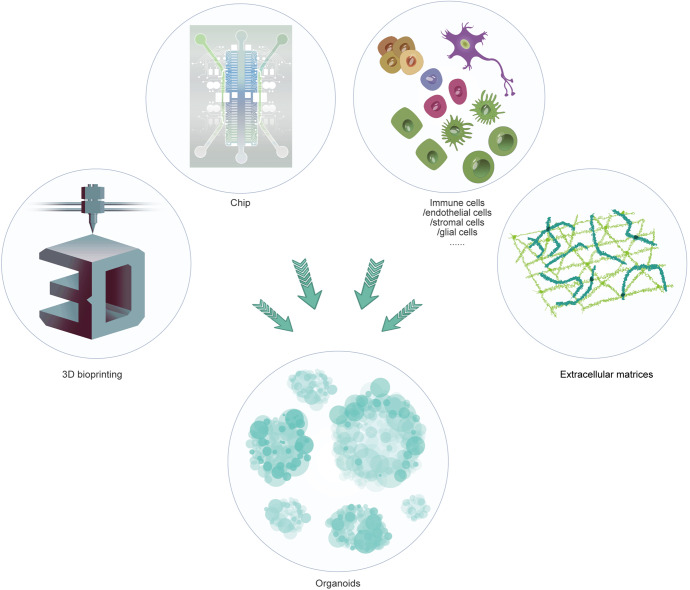
Advancements in organoid cellular and functional integrity. Advancement of organoid models: improving organoid complexity by adding mesenchymal cells, immune cells, endothelial cells, nerve cells, etc.; combining organoids with 3D bioprinting, biological materials and chip technology to develop organoid models that closely resemble the physiology of the human digestive system

Although organoids have been widely used in various fields, the organoids currently being produced are mainly epithelial systems. These simple components leave many studies unable to truly simulate the in vivo response, and the response of the organ should be the result of the mutual influence of various cells. Many drug effects on human tumors are often related to their microenvironment; for example, coculture of human PDAC PDOs and cancer-associated fibroblasts (CAFs) increases resistance to gemcitabine.^423^ In addition, the optimal composition of matrigel used for organoids is still unclear, severely limiting clinical application. In addition, the current culture systems are very different from the in vivo environment, and it is difficult to provide sufficient oxygen and nutrient supply for organoids growth. Further integration of microenvironmental components would allow digestive organoids to represent in vivo physiology more faithfully. Therefore, the major advances in organoids are more cellular components, more specific matrix components, and more suitable culture methods (Fig. 4).

### Construction of microenvironment and exploration of interactions between organs

**Fig. 5:**
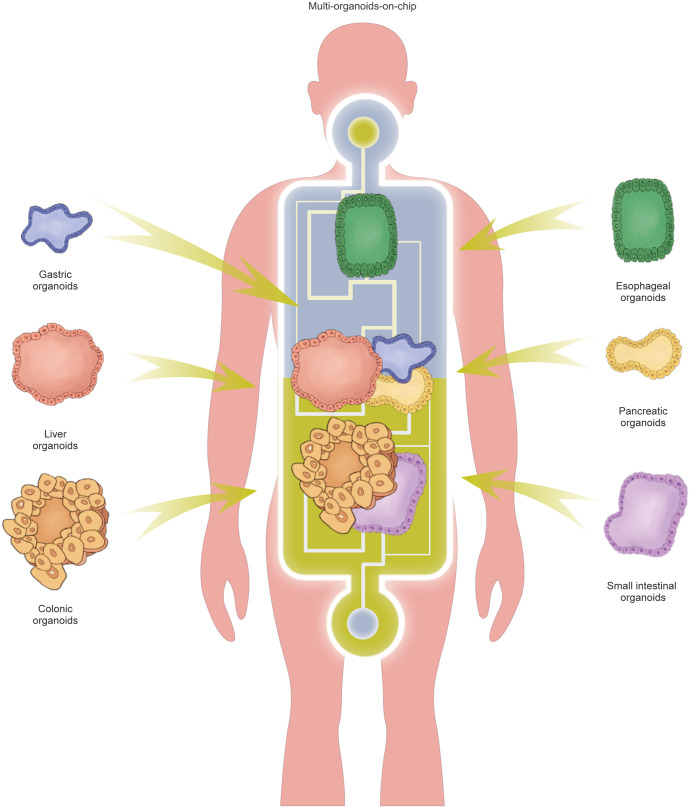
Future trends in the development of multi-organoid models. Organoids of the esophagus, pancreas, stomach, and intestines can be generated in vitro and combined to form a multi-organoid model on a microarray to test multiorgan diseases and cancer metastasis as well as drug toxicity and efficacy

The digestive organ’s microenvironment is a complex, dynamic, and multicellular system that features ongoing interactions among its components. However, existing organoid systems contain predominantly epithelial cells, low levels of maturation and cellular complexity.^464^ Importantly, building fully complex, well-functioning organoids enables more realistic studies of complex diseases such as cancer, syndromic diseases, and pathogen (bacterial and viral) infections. There are two ways through which organoid culture systems can achieve sufficient cellular complexity to recapitulate the function of the corresponding organs. In iPSC-derived organoids, the emergence of target cells can be induced by introducing transcription factors or exogenous mediators to promote organoid maturation. Induction of enteroendocrine cell (EEC) development can be realized by transient expression of NEUROG3 in iPSC-derived organoids.^465^ Intestinal microfold cells (M cells) are a dynamic epithelial cell lineage that initiates intestinal mucosal immunity; they have been successfully differentiated by adding activator of NF-κB ligand receptor (RANKL) to the culture medium, which induced the formation of M cells in organoid cultures by upregulating the transcription factor SpiB.^466^ TGF-β1 and Sonic Hedgehog (SHH) signaling regulate the appearance of muscularis mucosa (MM) of human gastric organoids derived from iPSCs.^467^ BMP activation drives the expression of villus tip genes, and loss of the Bmpr1a receptor in human organoids reduces the expression of such genes.^468^ When cell atlas and organoid technologies were combined to discover the effects of stem cell niche characteristics on mesenchymal-derived cells, the results suggested that the homeobox transcription factor CDX2 was required for epithelial and mesenchymal compartmentalization, while NRG1 promoted intestinal stem cell maturation.^355^ The enteric nervous system (ENS), part of the autonomic nervous system, has remarkable neurotransmitter diversity and complex cytoarchitecture. Human PSC-derived neural crest cells (NCCs) or blood vessels can be individually introduced to HIOs and assembled with them at appropriate times to form a functional enteric nervous system (ENS) and blood vessels in the organoids.^469^ Refining existing intestinal coculture systems is another way to achieve the desired grade of organoid maturity and cellular complexity. These coculture systems can be formed with organoids through two types of strategies. In the reconstituted model, organoids are cultured in extracellular matrix domes, and other cells are immersed in culture medium or the bottom layer of Transwell plates. In holistic native tumor microenvironment (TME) models without reconstitution, organoids from digested tissue can be mixed with collagen and injected into microfluidic systems or other culture devices to preserve the epithelium and its microenvironment in vitro. Complex cocultures of immune cells with organoids provide a versatile tool to study bidirectional interactions that support the delicate balance of digestive homeostasis. Coculture with innate lymphocytes (ILCs) will show how different subsets of ILCs affect intestinal epithelial barrier integrity and regeneration.^470^ When cocultured with interleukin 2 (IL-2)-secreting immune cells, human PSC-derived intestinal organoids rapidly mature into adult intestinal epithelial cells and increase gut-specific functional activity.^471^ Coculture of human intestinal stem cell-derived enterocytes and human monocyte-derived macrophages improves intestinal cell barrier function and maturation.^472^ Some subtypes, such as PTGER4^+^ intestinal macrophages, support the intestinal stem cell niche in organoids.^473^ A recent study went a step further, describing a platform for integrating patient-specific mature lymph node antigen-presenting cells into organoids for adaptive immunity.^474^ In addition to coculture of immune cells, other cell types have been extensively explored in recent decades, and a series of fruitful analyses have been performed on the degree of phenotypic mimicry and molecular mechanistic replication of in vivo function after coculture. The mesenchymal system is generally thought to be involved in stem cell maintenance. Coculture of AdSC-derived small intestine with intestinal subepithelial myofibroblasts (ISEMF) allows long-term small intestine culture in the absence of certain growth factors required for organoid culture.^475^ The pericryptal CD34^+^Gp38^+^αSMA^-^ mesenchymal cells are the major intestinal producers of niche factors that promote the maintenance of stem cell populations in gut organoids.^476,477^ The WNT-dependent PDAC organoids grow independently of exogenous WNTs when cocultured with patient-derived CAFs.^174^ In Rspo-free medium, the growth of gastric organoids is supported as long as they are cocultured with stromal cells.^478^ Organoid cocultures were used to recapitulate portal mesenchymal cell architecture, including a periportal mesenchymal cell subset that acts as a rheostat to regulate ductal cell proliferation.^479^ CD90^+^ mesenchymal cells may enhance stem cell function due to the presence of L-arginine in the organoid and mesenchyme coculture systems, acting through mTORC1 to stimulate Wnt2b secretion.^480^ Pancreatic tumor spheroids were cocultured with pancreatic stellate cells in microchannels so that fibroblast activation could be observed.^481^ Human PDAC organoids and mouse CAFs were cocultured to identify heterogeneity within CAFs in order to guide therapy.^482^ The enteric nervous system is composed of many enteric glial cells (EGCs) and neurons, which are key components of the intestinal stem cell niche in regeneration and disease; these fibrillary acidic protein (GFAP)^+^ EGCs express multiple Wnt ligands and promote self-renewal of LGR5^+^ ISCs in organoid and glia coculture systems.^483^ Coculture with neurons similar to myofibroblasts enhances the growth of the organoids.^484,485^ Coculture of intestinal organoid monolayers with differentiated adipocytes induces reciprocal amplification of inflammatory responses.^486^ Organoids are cocultured with vascular endothelial cells and pericytes to form vascular organoids, providing hope for the further development of organoid model systems. Endothelial cells were added to a miniature device in which isolated intestinal organoid cells had been found to communicate with intestinal epithelial cells and form a blood vessel-like network.^224^ Vascularization can be implemented by transplantation into mice or by using microfluidic devices to connect vascular networks to intestinal organoids from a highly vascularized tissue, for example, in the case of human PSC-derived intestinal organoids implanted in mouse kidneys.^487^ Holistic native TME models without reconstitution favor immunotherapeutic effects or organoid transplantation for regenerative treatment of bowel disease. Tumor tissue and a mixture of stromal fibroblasts and immune components were cocultured using an air-liquid interface organoid culture system, and heterogeneous T cells from the original tumor were also preserved in these cultures.^223^ An in vitro model in which organoids from clinical CRC tissue were mixed with TILs from patients and *F. nucleatum* showed that exposure to *F. nucleatum* increased sensitivity to PD-L1 blockade.^488^ Organoids with diverse cellular components will advance organoid technology for studying the physiological and pathological functions of the digestive system. Another notable direction of progress is the establishment of a multi-organoids model, which is interesting and necessary because of the interaction of multiple organs in the process of disease development. Methods have recently been developed for the fusion of organoids and coculture of multiple organoids. For example, heart-lung-liver organoid models have been assembled in a modular manner through microengineering.^489^ A new protocol for generating integral multiorgan structures of the digestive system has also been reported, i.e., the fusion of two PSC-derived spheroids that correspond to the anterior and posterior gut to the creation of hepato-biliary-pancreatic organoids that establish boundary interactions between two spheroids without any extrinsic factors.^490^ If the timing and composition of the medium are tightly controlled, different types of organoids can be made to grow independently on the microfluidic chip but still communicate with each other. For example, fully functional tissues have been generated in microfluidic chip culture systems via the assembly of multiple organoids.^491^ Multilineage organoid models integrate cardiac and gut features arising from the aggregation of single mesodermal progenitors.^492^ Paracrine signals between adjacent tissues influence fate decisions of target organs during embryogenesis. Multi-organoids models can be used as in vitro models to simulate the effects of paracrine inputs from surrounding tissues on developmental and cellular heterogeneity during organ development. Multi-organoids model developed with microfluidic arrays were used to coculture stem cell-derived liver, intestine and stomach organoids; paracrine factors produced by intestinal organoids were detected to affect the expression of bile acid synthetase (CYP7A1) in liver organoids, which verified the interorgan interaction.^226^ Multiorgan metabolic diseases characterized by dynamic interactions between different organs, such as type 2 diabetes mellitus (T2DM), can be replicated through coculture interactions between different organoids. With the help of a microfluidic system consisting of two separated regions connected by a microchannel network, coculture systems can be established between liver and pancreas organoids derived from human iPSCs, recapitulating the physiological and pathological conditions of the human liver-islet axis and facilitating analytical work regarding this axis in health and disease.^493^ By integrating technologies such as microarray technology and multiple digestive system organoids, such as esophageal/gastric/liver/pancreatic/gut organoids, into in vitro models to bring them closer to human tissue structure and function, these advanced models can further bridge the gap between in vitro systems and animal experiments (Fig. 5).

### Optimization of culture conditions

Improvements in cultivation are another aspect in which progress has been made recently. These improvements have mainly been in two directions: replacing the extracellular matrix and improving organoid culture systems. On the one hand, a large number of current organoid systems rely on animal-derived extracellular matrix (mainly stroma and basement membrane extract (BME)), a protein mixture extracted from Engelbreth-Holm-Swarm mouse sarcoma.^494^ Matrigel/BME has no unified standard formula and shows batch-to-batch variability, which is a major roadblock for the translation of this methodology into the clinic, limiting its applicability to personalized medicine.^495^ Emerging matrigel-free methods for the culture of organoids can overcome these limitations; examples of matrigel-free approaches include decellularized ECM collagen, engineered ECM proteins and synthetic polymer hydrogels.^496^ Decellularized ECM from human or animal donors can accurately recapitulate native structure, ECM composition and vascularization during organ development, and decellularized ECM has been used to culture enteroids and HIOs derived from PSCs.^63,497^ Collagen I, a natural protein commonly obtained from pigs, has been used to culture human intestinal organoids.^498^ Synthetic hydrogels such as PDMS are attractive due to their controllable mechanical properties, functions, and erosion rates and are currently used to create organ-on-a-chip microfluidic chambers.^499^ Other alternatives, such as a recently developed synthetic hydrogel scaffold for use with polyethylene glycol (PEG), have shown similar results to matrigel in supporting organoid growth.^500^ However, these synthesized hydrogels have difficulty accurately reproducing the physicochemical properties of ECM in vivo, and the material can adsorb small hydrophobic molecules, which is disadvantageous in that it hinders drug discovery.^501^ There is a growing need to design and manufacture biosynthetic materials that mimic ECM function and preserve specific cellular properties while maintaining stem cell differentiation.^502^ Matrigel formulas made of peptides and recombinant proteins produced by genetically engineered organisms are attractive for organoid culture because their mechanical and chemical properties can be independently altered.^503^ Overall, the prospect of programmable recombinant protein synthesis materials has certain advantages for functionalizing the binding of biologically relevant cellular proteins or peptides, enabling the artificial ECM to reproduce the dynamic properties (erosion rate, viscoelasticity, and degradation sensitivity) as well as the correct chemical composition of the natural ECM. This artificial system with independently adjustable chemical and mechanical properties has been used to provide appropriate development and growth for various kinds of organoids. Another area that is being refined is organoid culture methods; using technologies such as 3D printing and microfluidics, problems such as structure and oxygen concentration can be solved to improve culture devices. 3D bioprinting technology includes loading tissue-specific cells into bio-inks to reconstruct various organ structures of the human body through layered printing technology.^504^ Another innovative technological combination involving organoids consists of “organoids-on-a-chip”. This system is a microfluidic device that grows cells and tissues in a continuous perfusion chamber. Organoids-on-a-chip can form a dynamic and controllable digestive system microenvironment. It is expected to recapitulate the physiology and pathology of living human organs in vitro and model organ-organ interactions, even serving as an alternative to animal models in the future.^505^ Bottom-up tissue engineering strategy building blocks can be used to generate organic cultures of multicellular superstructures, and those models may also permit the epithelium to be cultured with other cells based on a 3D structure with a type I collagen coating on a thin Matrigel base that mimics the morphology of the gut.^506^ Using the surfaces of tissue-engineered scaffolds to create crypt-like structures, intestinal stem cells have been induced to form tubular epithelial cells with accessible lumens and a spatial arrangement of crypt- and villus-like domains similar to those observed in vivo. By connecting an external pump system, the micro-scale intestinal tube can be perfused and the dead cells can be continuously cleared of, extending tissue life by weeks.^224^ In the absence of externally applied biochemical gradients, several recent approaches have created macroscopic organoids resembling periodic crypt-villus structures of native intestinal epithelium, using physiologically precise patterns and localizations conferred only by tissue geometry.^99^ In addition, robotic pipelines using traditional machines require fewer cells to automate work, illustrating that automation helps simplify and harmonize steps in the workflow.^507^ To date, fully automated 3D culture protocols for kidney organoids using liquid-handling robots have been established.^508^ In the future, more integrated technologies are required to combine all test conditions in one organoids culture and ultimately generate organoids with complete structure and function, reproduce the physiological functions of target organs, and further advance the state of clinical treatments.

## Conclusion and perspective

**Fig. 6:**
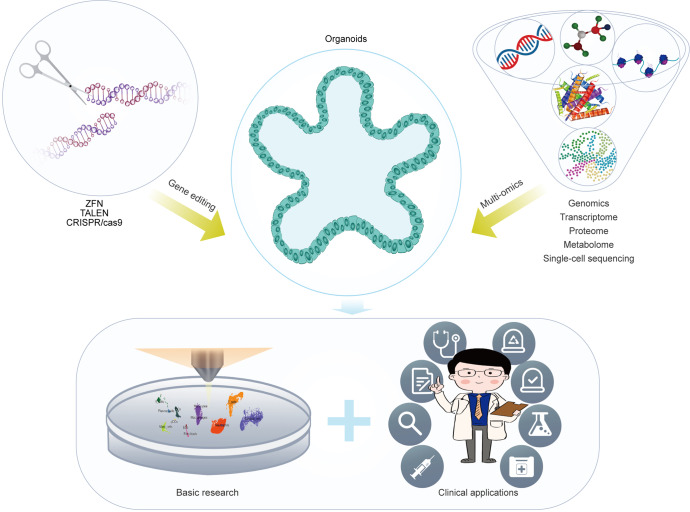
The prospect of combining high-technology approaches and digestive organoids. Gene editing and omics (epigenetics, transcriptomics, proteomics, metabolomics, and single-cell sequencing) technologies are used in combination with organoid models to systematically and comprehensively analyze molecular, cellular, and histological changes mediated by gene mutations or environmental interventions. Starting from basic research, the in vivo phenotypes and molecular targets corresponding to organoids under different conditions are continuously explored to guide the selection and testing of clinical treatment strategies

Digestive organoids are 3D in vitro cultured organ models with similar structures that contain multiple cell types, including stem cells and differentiated cells.^509^ These organoid models can overcome the genetic variability of traditional cell culture systems and the pitfalls of interspecies dissimilarity between humans and mice, and they have the potential to provide personalized therapy for a wide range of patients, even at various preclinical stages. Thus, human organoids represent an in vitro testing model for drug screening or gene/cell therapy and serve as a bridge between in vitro and in vivo systems.^510^ In particular, the large-scale application of existing 3D PDO cultures has transformed in vitro cancer biology and enabled the construction of large tumor biobanks to capture the mutational diversity and histology of human cancers.^40^ To date, studies of digestive organoid research have covered the modeling of digestive system disease, establishment of organoid biobanks, transplantation of organoids into the injured epithelium for regeneration, repair of disease-related defects via gene editing, high-throughput drug screening, and characterizing the responses to bacterial and viral infection.^511^ However, although organoids have yielded many useful results in various fields of digestive system research, certain limitations of current organoids have also created bottlenecks that are currently insurmountable, requiring more breakthroughs. Emerging technologies, such as the application of CRISPR-Cas9 technology to silence or activate specific genes in organoids, will open new doors for understanding the role of specific genes in human digestive system disease.^139,512–514^ The distribution and function of target genes during digestive development or progression of disease can be detected in real time by gene knockin fluorescent labeling in iPSCs.^514,515^ Single-cell RNA sequencing (scRNA-seq) methods have changed the paradigm of biomedical science by analyzing cellular heterogeneity from multivariate data.^516^ scRNA-seq of organoids has allowed certain rare or abnormal cell types to be identified, and these findings can be detected in individual organoids.^98,517^ By deepening the combination with these new technologies, gene editing and omics (epigenetics, transcriptomics, proteomics, metabolomics, and single-cell sequencing), one can systematically and comprehensively analyze changes in molecular, cellular, and organizational structures mediated by gene mutations or environmental interventions, facilitating future basic research and clinical applications (Fig. 6). Additional microfluidic devices are moving from proof of concept to commercial tools, and easy-to-use devices have facilitated the development of high-performance sensors that can record oxygen levels, transepithelial resistance and various experimental data in real time.^314^ We anticipate that the implementation of organoids, standard protocols and sophisticated devices will help the scientific community discover new mechanisms of action of pathology in the digestive system and identify biomarkers and follow-up targets for precision therapy for subsequent clinical trials.

In conclusion, given the incomplete cellular composition and function of various digestive organoids, further optimization is required to enhance their application in disease modeling and personalized medicine. When organoids are derived from tissue or cells that have been directly extracted from diseased patient samples, they preserve the genetic and pathological information of the original tissue, and their 3D physiological structure and cellular composition remain relatively close to the corresponding human organ, opening a vital avenue for innovation in precision and personalized medicine. Combined with other bioengineering methods and omics techniques, organoid models will undoubtedly play an invaluable role in preclinical and clinical research.
